# Structural cortical network reorganization associated with early conversion to multiple sclerosis

**DOI:** 10.1038/s41598-018-29017-1

**Published:** 2018-07-16

**Authors:** C. Tur, A. Eshaghi, D. R. Altmann, T. M. Jenkins, F. Prados, F. Grussu, T. Charalambous, A. Schmidt, S. Ourselin, J. D. Clayden, C. A. M. G. Wheeler-Kingshott, A. J. Thompson, O. Ciccarelli, A. T. Toosy

**Affiliations:** 1 Queen Square MS Centre, Department of Neuroinflammation, UCL Institute of Neurology, Faculty of Brain Sciences, University College of London (UCL), London, WC1B 5EH UK; 2 Centre for Medical Image Computing (CMIC), Department of Computer Science, University College London (UCL), London, WC1E 7JE UK; 3 Medical Statistics Department, London School of Hygiene and Tropical Medicine, University of London, London, UK; 4 Translational Imaging Group, Centre for Medical Image Computing, Department of Medical Physics and Biomedical Engineering, UCL, London, WC1E 7JE UK; 5 Department of Psychiatry (UPK), University of Basel, Basel, Switzerland; 6 UCL Great Ormond Street Institute of Child Health, UCL, London, WC1N 1EH UK; 7 Brain MRI 3T Research Center, C. Mondino National Neurological Institute, Pavia, Italy; 8 Department of Brain and Behavioral Sciences, University of Pavia, Pavia, Italy; 9 National Institute for Health Research University College London Hospitals Biomedical Research Centre, London, UK

## Abstract

Brain structural covariance networks (SCNs) based on pairwise statistical associations of cortical thickness data across brain areas reflect underlying physical and functional connections between them. SCNs capture the complexity of human brain cortex structure and are disrupted in neurodegenerative conditions. However, the longitudinal assessment of SCN dynamics has not yet been explored, despite its potential to unveil mechanisms underlying neurodegeneration. Here, we evaluated the changes of SCNs over 12 months in patients with a first inflammatory-demyelinating attack of the Central Nervous System and assessed their clinical relevance by comparing SCN dynamics of patients with and without conversion to multiple sclerosis (MS) over one year. All subjects underwent clinical and brain MRI assessments over one year. Brain cortical thicknesses for each subject and time point were used to obtain group-level between-area correlation matrices from which nodal connectivity metrics were obtained. Robust bootstrap-based statistical approaches (allowing sampling with replacement) assessed the significance of longitudinal changes. Patients who converted to MS exhibited significantly greater network connectivity at baseline than non-converters (p = 0.02) and a subsequent connectivity loss over time (p = 0.001–0.02), not observed in non-converters’ network. These findings suggest SCN analysis is sensitive to brain tissue changes in early MS, reflecting clinically relevant aspects of the condition. However, this is preliminary work, indicated by the low sample sizes, and its results and conclusions should be treated with caution and confirmed with larger cohorts.

## Introduction

GM atrophy is a key determinant of long-term disability in MS^1–3^. However, the intermediate steps linking brain tissue damage to accrual of disability in MS are not well understood and therefore cannot be effectively tackled by treatments or used to build accurate predictive models of clinical progression. In this context, it has been proposed that the disruption of functional and structural brain networks may be related to these intermediate processes leading, eventually, to physical and cognitive disability in MS^4^.

Among the types of studied brain networks, there are structural covariance networks (SCNs), which are based on the anatomical similarity, at the group level, between different cortical areas^5–8^. Cortical areas sharing common functions with evolutionary shared development have greater similarity for macroscopic features than non-functionally related regions^9^. Thus, the study of the changes in the patterns of morphological similarity in the cortex has emerged as a useful tool to examine the link between structural damage and functional consequences^10^.

SCNs are constructed from the statistical covariance between cortical regions in terms of any morphological variable, such as cortical thickness, cortical surface or cortical volume. However, SCNs built based on the statistical covariance of thicknesses of different cortical regions are most frequently studied. Thus, the nodes of these networks are the different brain areas, and the edge connecting any two of these nodes is determined as the correlation in thicknesses between cortical areas forming the two nodes. Because SCNs are strongly influenced by congenital and developmental functional relationships between cortical areas, but are also susceptible to acquired damage in the cortical GM, the investigation of SCN can provide information complementary to functional and diffusion-based connectivity analyses^6,7,11^.

SCNs have also the potential to provide information complementary to more classical univariate analyses of structural damage, such as cortical thinning. Covariance network analysis accounts for inter-regional correlations in cortical thickness data and allows us to estimate the actual impact of such correlations on disease progression. This implies that SCN analysis is able to better evaluate the complexity of human brain cortex structure than classical univariate approaches, where relationships between cortical regions are not taken into consideration. In fact, SCN analysis has been applied to several neurodegenerative disorders, revealing neurodegeneration at the network level that might be missed at regional-level^12^.

However, despite the potential of SCN analysis to unveil new mechanisms of neurodegeneration, the longitudinal assessment of SCN dynamics and their pathological relevance have not yet been explored.

We hypothesised that longitudinal SCN analyses in patients at the earliest stages of MS may provide complementary information to conventional cortical GM thickness analysis, revealing important –and otherwise hidden– features of GM dynamics. Our aims were to characterise the longitudinal changes in SCN parameters in the early CIS, comparing the behaviour of SCNs of CIS patients and age-matched healthy controls, and importantly, to investigate the clinical relevance of SCNs, through assessing SCN changes in those who converted to clinically definite MS within the first year. Our aims were achieved applying robust, computationally intensive bootstrapping-based statistical methods to obtain data-driven estimations of confidence intervals of our network parameters^13^, which allowed us to make unbiased statistical inferences. Of note, given the particular group-level nature of SCN metrics, such statistical inferences would not have been possible using more conventional statistical approaches.

## Results

### Descriptive statistics

For this study, we included 21 patients (18 female, mean age [standard deviation, SD]: 32.24 [6.18] years) and 7 healthy controls (HCs) (5 female; 30.14 [3.44] years). All subjects included in this study belong to a cohort of 28 CIS patients and 10 HCs, whose clinical and demographic features have been previously reported^14–17^. We excluded: two HCs because they only had baseline data; one HC and four CIS patients because of the low quality of the cortical thickness segmentation; and three CIS patients because they did not attend the last follow-up and they had also missed at least one more time point, implying that a single imputation method would have not provided reliable enough information.

During follow-up, nine patients presented a second clinical attack suggestive of MS. These patients were diagnosed with clinically definite MS (called ‘clinical MS converters’). Three additional patients presented new (asymptomatic) lesions, fulfilling the criteria for dissemination in space (DIS) and time (DIT). Thus, the nine patients with a second attack plus the three patients fulfilling DIS and DIT criteria through MRI fulfilled the 2010 McDonald criteria for MS^18^ (Table 1).

**Table 1:** Demographical, clinical and MRI characteristics of the study cohort.

	All HCs	All CIS	HCs vs. CIS, p-value	CIS-CIS^a^	CIS-MS^b^	CIS-CIS^a^ vs. CIS-MS^b^, p-value
N finally included	7	21	—	12	9	—
Age at study onset, mean (SD)	30.143 (3.436)	32.238 (6.180)	p = 0.405^e^	30.917 (5.368)	34 (7.053)	p = 0.268^e^
No. females	5	18	p = 0.393^f^	10	8	p = 0.719^f^
Cortical thickness at baseline, mean (SD)	2.751 (0.058)	2.713 (0.135)	p = 0.748^g^	2.748 (0.075)	2.667 (0.184)	p = 0.508^g^
Cortical thickness at 12 m, mean (SD)	2.726 (0.052)	2.715 (0.087)	p = 0.367^g^	2.750 (0.060)	2.672 (0.100)	p = 0.910^g^
Lesion volume at baseline, median (range)	—	1.525 mL (0 to 16.199 mL)	—	0.534 mL (0 to 3.516 mL)	5.260 mL (1.525 to 16.199 mL)	p = 0.001^e^
Lesion volume at 3 months, median (range)	—	1.828 mL (0 to 18.536 mL)	—	0.631 mL (0 to 2.918 mL)	6.113 mL (1.828 to 18.536 mL)	p = 0.001^e^
Lesion volume at 6 months, median (range)	—	2.101 mL (0 to 17.816 mL)	—	0.571 mL (0 to 2.716 mL)	5.397 mL (1.521 to 17.816 mL)	p = 0.002^e^
Lesion volume at 12 months, median (range)	—	2.918 mL (0 to 17.038 mL)	—	0.756 mL (0 to 4.891 mL)	5.322 mL (2.246 to 17.038 mL)	p = 0.005^e^
DIS^c^ at baseline, no.	—	10	—	4	6	p = 0.130^f^
DIS^c^ at 3 months, no.	—	10	—	4	6	p = 0.130^f^
DIS^c^ at 6 months, no.	—	9	—	3	6	p = 0.078^f^
DIS^c^ at 12 months, no.	—	12	—	5	7	p = 0.094^f^
DIS^c^ and DIT^c,d^ at baseline, no.	—	0	—	0	0	p > 0.99^f^
DIS^c^ and DIT^c,d^ at 3 months, no.	—	3	—	0	3	p = 0.031^f^
DIS^c^ and DIT^c,d^ at 6 months, no.	—	6	—	2	4	p = 0.202^f^
DIS^c^ and DIT^c,d^ at 12 months, no.	—	9	—	3	6	p = 0.058^f^

^a^Patients who did not have a second attack during the follow-up; ^b^patients who had a second attack during the follow-up; ^c^according to the 2010 Revisions of the McDonald Criteria; ^d^patients did not undergo a whole-brain T1-weighted scan after Gadolinium injection. Therefore, it was not possible to assess whether gadolinium-enhancing lesions were coexisting with non-gadolinium-enhancing lesions, at any time point; ^e^two-sample t-test; ^f^chi-square test; ^g^linear regression adjusting for age, gender and T2 lesion load. *Abbreviations:* CIS: clinically isolated syndrome; DIS: dissemination in space; DIT: dissemination in time; HCs: healthy controls; SD: standard deviation.

### Cortical thickness and other structural imaging data analyses

At baseline, mean (SD) cortical thickness was 2.713 (0.135) mm in patients, and 2.751 (0.058) mm in controls. At follow-up, mean (SD) cortical thickness was 2.715 (0.087) mm in patients and 2.726 (0.052) mm in HCs. In patients, median lesion loads at baseline and follow-up were 1.525 mL (range: 0 to 16.199 mL) and 2.918 mL (range: 0 to 17.038 mL). The main locations of the MS lesions are shown in Supplementary Table 1. After adjusting for age, gender and T2 lesion load, no significant differences in mean cortical thickness were observed between CIS patients and HCs and baseline or at one-year follow-up (p = 0.748 and 0.367, respectively). Over time, neither CIS patients: −0.0003 mm/month (95% CI: −0.003 to 0.003 mm/month), p = 0.822, nor HCs: −0.002 mm/month (−0.004 to 0.0004 mm/month), p = 0.338, showed a significant change in mean cortical thickness over time. No differences between these rates of change were observed either (p = 0.46).

Similarly, no differences in cortical thickness were observed between patients with and without early conversion to MS at baseline or follow-up (p = 0.5079 and 0.9103, respectively), after adjusting for age, gender and T2 lesion load. Additionally, none of the two patient groups showed a significant change in cortical thickness over one year: CIS with conversion to MS: 0.001 (−0.005 to 0.007), p = 0.655; CIS without conversion to MS: −0.001 (−0.005 to 0.004), p = 0.586. Besides, these rates of change were not significantly different from each other (p = 0.4842).

### Structural cortical network analysis

#### All CIS patients vs. HCs

**Figure 1:**
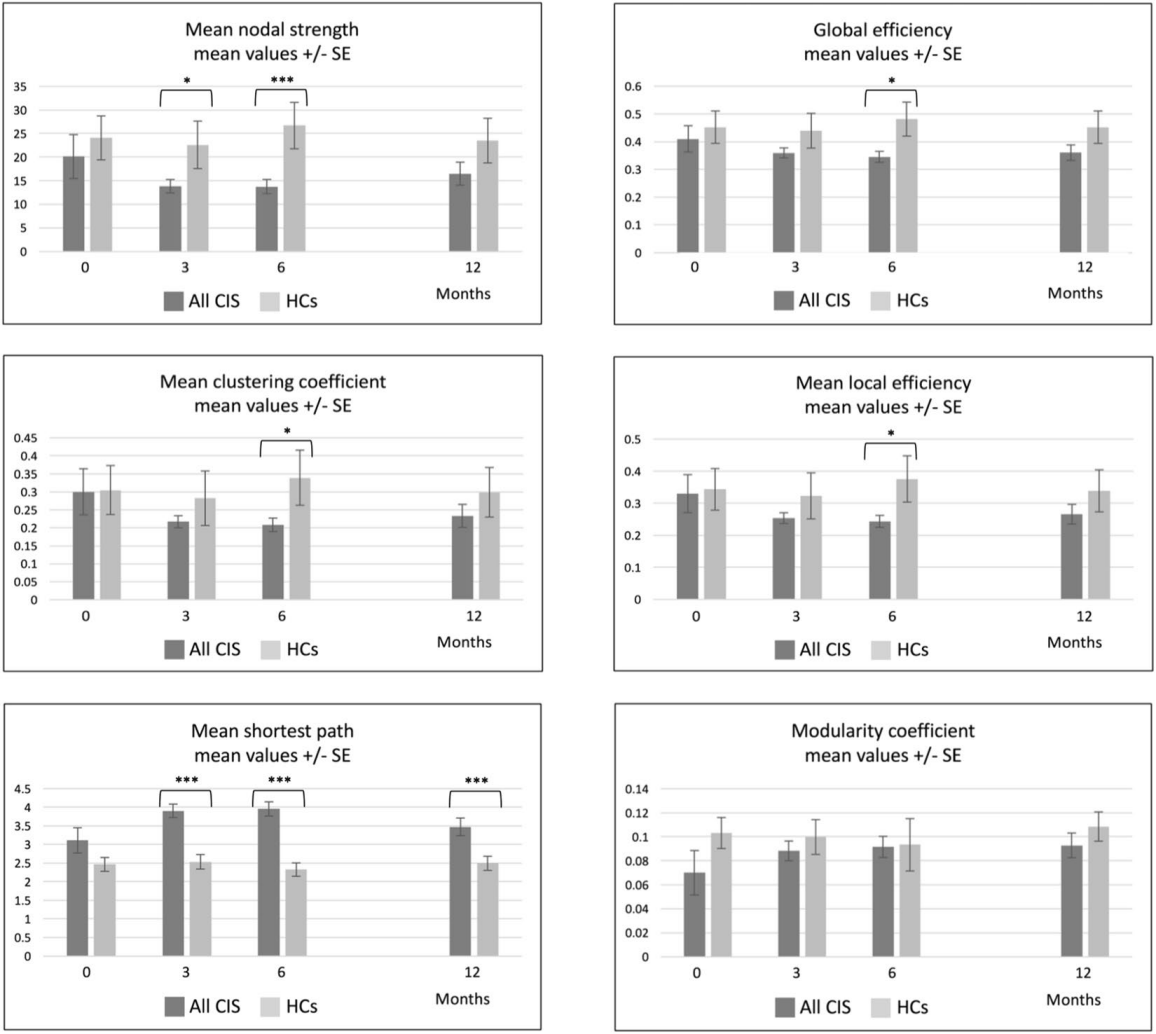
Network metrics over time in all CIS patients and HCs. This figure shows the evolution of network metrics over time for patients’ and controls’ networks, with the bootstrap standard errors. Although at follow-up only the mean shortest path was significantly different between patients’ and controls’ networks, at six-month follow-up, most of the metrics were significantly different between the two networks; *p < 0.05; **p < 0.01; ***p < 0.001.

At baseline, no differences were found in any of the metrics between all CIS patients and HCs (Fig. 1 and Supplementary Figure 1).

When looking at the changes over time, CIS patients’ network tended to show an overall decrease in connectivity and significant differences in several metrics were observed between patients’ and controls’ networks at 3 and, especially, at 6 months of follow-up: mean nodal strength (p < 0.001), mean clustering coefficient (p = 0.01), mean shortest path (p < 0.001), global efficiency (p = 0.01) and local efficiency (p = 0.01) (Fig. 1). Yet none of the 12-month longitudinal changes in either patients’ or controls’ networks reached statistical significance (Table 2).

**Table 2:** One-year changes in SCN parameters, in CIS patients and HCs.

	CIS patients (all)	HCs	Patients vs. HCs, estimated p-value
***1. Measures of nodal connectivity***			
**Mean nodal strength, estimated value (bootstrap-based 95% CI), p-value**			
Change from baseline to 1 year	−3.62 (−12.29 to 2.19), p = 0.3	−0.58 (−3.37 to 2.03), p = 0.7	0.50
**Mean clustering coefficient, estimated value (bootstrap-based 95% CI), p-value**			
Change from baseline to 1 year	−0.06 (−0.19 to 0.02), p = 0.15	−0.01 (−0.05 to 0.04), p = 0.8	0.30
***2. Measures of nodal distance***			
**Mean shortest path, estimated value (bootstrap-based 95% CI), p-value**			
Change from baseline to 1 year	0.36 (−0.13 to 0.89), p = 0.2	0.03 (−0.06 to 0.13), p = 0.6	0.25
**Global efficiency, estimated value (bootstrap-based 95% CI), p-value**			
Change from baseline to 1 year	−0.05 (−0.14 to 0.02), p = 0.15	−1.46 * 10^−4^ (−0.03 to 0.03), p = 0.99	0.20
**Mean local efficiency, estimated value (bootstrap-based 95% CI), p-value**			
Change from baseline to 1 year	−0.06 (−0.17 to 0.01), p = 0.15	−0.005 (−0.05 to 0.04), p = 0.9	0.25
***3. Measures of network organisation***			
**Modularity coefficient (bootstrap-based 95% CI), p-value**			
Change from baseline to 1 year	0.02 (−0.01 to 0.06), p = 0.2	0.0054 (−0.03 to 0.04), p = 0.7	0.60

Comparison between CIS patients and HCs was made looking at the 95% CI. See main text for full details on the methods used. Significant p-values are indicated with bold letters. *Abbreviations:* CI: confidence interval; CIS: clinically isolated syndrome; FU: follow-up; HCs: healthy controls.

Supplementary Figure 2 shows the bootstrap distributions used to ascertain the statistical significance of the 12-month changes in network parameters and, as mentioned, no differences between groups were observed. Supplementary Table 2 shows the point estimates and bootstrap-based 95% CIs for baseline and 12-month network parameters: only the mean shortest path was significantly different (i.e. greater, p < 0.001) in patients than controls, at 12-month follow-up.

#### CIS patients with vs. without clinical conversion to MS

**Figure 2:**
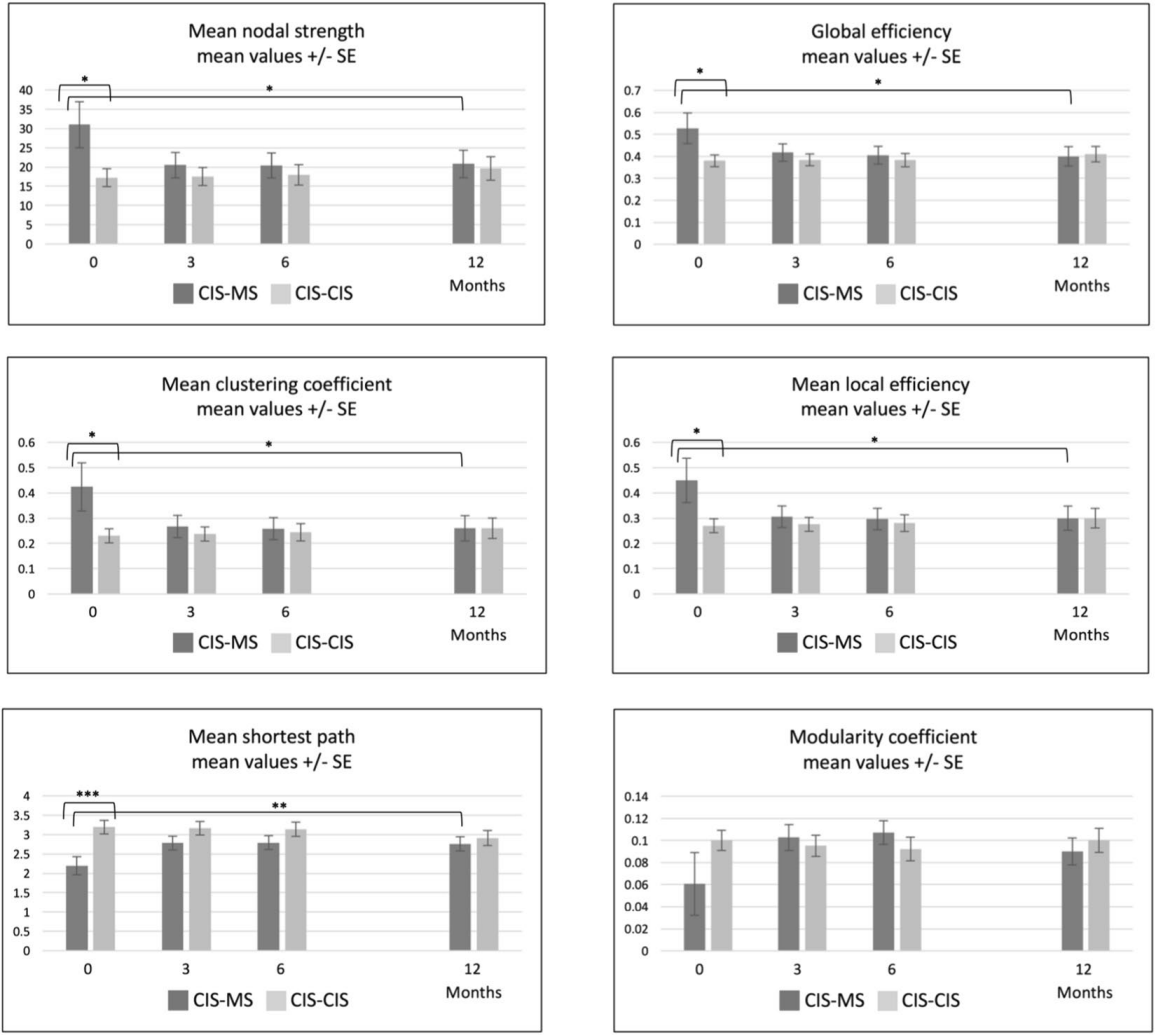
Network metrics over time in MS converters and non-MS converters. This figure shows the evolution of network metrics over time for MS converters and non-MS converters, with the bootstrap standard errors. *p < 0.05; **p < 0.01; ***p < 0.001. Please look at Table 3 for more details.

At baseline, the network of CIS patients who later clinically converted to MS during the follow-up showed features of greater nodal connectivity, i.e. significantly greater mean nodal strength (p = 0.02) and clustering coefficient (p = 0.02), and features of better network *integration*, i.e. lower mean shortest path (p < 0.001) and greater global (p = 0.02) and local (p = 0.02) efficiency, than the network of non-converters, who showed similar values to HCs’ network (Fig. 2).

Over one year, clinical MS converters’ network showed significant changes in all metrics related to nodal connectivity: mean nodal strength decreased by 10.18 units (bootstrap-based 95% CI: −16.22 to −0.87), p = 0.02; mean clustering coefficient decreased by 0.16 units (−0.27 to −0.01), p = 0.02; and all metrics related to nodal distance: both global (−0.12 (−0.20 to −0.02), p = 0.01) and local efficiency (−0.15 (−0.25 to −0.01), p = 0.02) decreased, whereas mean shortest path increased (0.56 (0.19 to 0.80), p = 0.001) over one year. Instead, non-converters network did not show any significant changes over time, except for the mean shortest path, which decreased over 1 year (p = 0.01), mirroring HCs’ network behaviour. The rates of change over time of the two networks were all strongly significantly different from each other (p-values from 0.01 to <0.001), except for the change in modularity coefficient (p = 0.5) (Table 3 and Fig. 2). Supplementary Figures 3 and 4 show the bootstrap distributions used to ascertain the statistical significance of the parameters. Supplementary Table 3 shows the point estimates and bootstrap-based 95% CIs for baseline and 12-month network parameters.

**Table 3:** One-year changes in SCN parameters, in CIS patients with and without clinical conversion to MS.

	Patients with clinical MS conversion	Patients without clinical MS conversion	Patients with vs. without clinical MS conversion, estimated p-value
***1. Measures of nodal connectivity***			
**Mean nodal strength, estimated value (bootstrap-based 95% CI), p-value**			
Change from baseline to 1 year	−10.18 (−16.22 to −0.87), **p** = **0.02**	2.39 (−1.03 to 7.52), p = 0.3	**0.01**
**Mean clustering coefficient, estimated value (bootstrap-based 95% CI), p-value**			
Change from baseline to 1 year	−0.16 (−0.27 to −0.01), **p** = **0.02**	0.03 (−0.02 to 0.11), p = 0.4	**0.01**
***2. Measures of nodal distance***			
**Mean shortest path, estimated value (bootstrap-based 95% CI), p-value**			
Change from baseline to 1 year	0.56 (0.19 to 0.80), **p** = **0.001**	−0.28 (−0.58 to −0.07), **p** = **0.01**	**<0.001**
**Global efficiency, estimated value (bootstrap-based 95% CI), p-value**			
Change from baseline to 1 year	−0.12 (−0.20 to −0.02), **p** = **0.01**	0.03 (−0.01 to 0.09), p = 0.2	**0.01**
**Mean local efficiency, estimated value (bootstrap-based 95% CI), p-value**			
Change from baseline to 1 year	−0.15 (−0.25 to −0.01), **p** = **0.02**	0.03 (−0.02 to 0.10), p = 0.3	**0.01**
***3. Measures of network organisation***			
**Modularity coefficient (bootstrap-based 95% CI), p-value**			
Change from baseline to 1 year	0.03 (−0.02 to 0.07), p = 0.5	0.01 (−0.02 to 0.03), p = 0.8 l	0.5

Comparison between patients with and without MS conversion was made looking at the 95% CI. See main text for full details on the methods used. Significant p-values are indicated with bold letters. *Abbreviations:* CI: confidence interval; CIS: clinically isolated syndrome; FU: follow-up; HCs: healthy controls.

**Figure 3:**
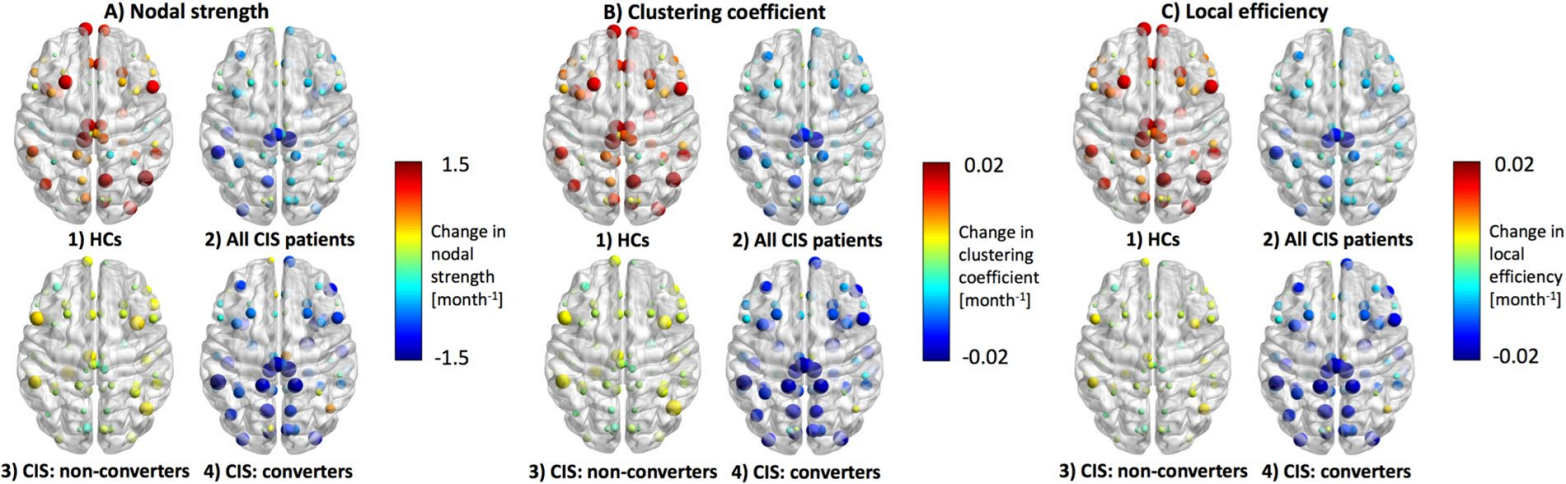
Estimated monthly changes in nodal metrics for all groups. This figure shows the estimated monthly changes in the three node-level metrics: (**A**) nodal strength, (**B**) clustering coefficient and (**C**) local efficiency. In this figure, the size of the nodes indicates the absolute value of the change: bigger nodes have greater changes, in either direction, and smaller nodes have changes close to zero. Instead, the color of the nodes indicates the direction of the change: *redder* nodes indicate positive changes (increase) and *bluer* nodes indicate negative changes (decrease). The behavior over time of all-CIS network differed from that of HCs’ network, yet these differences did not reach statistical significance (see Table 2). This different behavior was mirrored by the two patient groups: whereas the non-converters’ network only showed minimal changes over time, the converters’ network showed an overall decrease in nodal strength, clustering coefficient and local efficiency. Of note, the regional distribution of nodes with greatest changes over time (pre- and post-central gyri and paracentral lobule, bilaterally) was similar for the all-CIS and the converters’ networks. *Abbreviations:* CIS: clinically isolated syndrome; HCs: healthy controls.

When analysing regional changes in those metrics that admit a node-level analysis, i.e. nodal strength, clustering coefficient and local efficiency, the changes in the network of patients with an early conversion to MS showed a very similar pattern to those network changes observed for all patients together. Instead, network changes of those who did not convert were similar to changes in HCs’ network (Fig. 3). Please see Table 4 for a summary of these results.

**Table 4:** Summary table of the differences between groups.

	All CIS vs. HCs	CIS with vs. without MS conversion	CIS with MS conversion vs. HCs	CIS without MS conversion vs. HCs
**Mean nodal strength at baseline**				
Baseline	No	**Yes**	No	No
Change over time	No	**Yes**	No	No
Follow-up	No	No	No	No
**Mean clustering coefficient**				
Baseline	No	**Yes**	No	No
Change over time	No	**Yes**	No	No
Follow-up	No	No	No	No
**Mean shortest path**				
Baseline	No	**Yes**	No	**Yes**
Change over time	No	**Yes**	**Yes**	**Yes**
Follow-up	**Yes**	No	No	No
**Global efficiency**				
Baseline	No	**Yes**	No	No
Change over time	No	**Yes**	**Yes**	No
Follow-up	No	No	No	No
**Mean local efficiency**				
Baseline	No	**Yes**	No	No
Change over time	No	**Yes**	No	No
Follow-up	No	No	No	No
**Modularity coefficient**				
Baseline	No	No	No	No
Change over time	No	No	No	No
Follow-up	No	No	No	No

This table indicates whether there were significant differences between groups (indicated as ‘Yes’) or not (indicated as ‘No’). *Abbreviations:* CIS: clinically isolated syndrome; HCs: healthy controls; MS: multiple sclerosis.

A post-hoc analysis comparing the network of patients who were diagnosed with McDonald MS (i.e. either they had a second clinical attack or fulfilled the criteria for DIS and DIT during the follow-up, N = 12) with the network of those not fulfilling the diagnosis of McDonald MS (N = 9) was also performed. This comparison revealed very similar results to those obtained with the comparison of clinical MS converters (N = 9) vs. non-clinical MS converters (N = 12), as shown in Supplementary Tables 4 and 5.

## Discussion

In this longitudinal study, we provide evidence, for the first time, of reorganisational changes in SCNs of CIS patients who experience an early conversion to clinically definite MS, in the absence of detectable cortical atrophy. These results suggest that SCN analysis may be more sensitive to those changes occurring in the cortical GM at the earliest stages of the disease than conventional, regression-type (average-based) approaches of structural data. Besides, these results indicate that SCN changes may be clinically relevant.

Although structural covariance networks and cortical thickness data are indirectly related^8^, structural networks reflect global aspects of the data that cannot be captured by conventional analyses of cortical thickness: SCNs reflect the associations between cortical regions, and their longitudinal evaluation hence reflects how these associations change over time, revealing hidden patterns in GM dynamics not observable with classical (linear regression-type) approaches^8^. However, despite numerous longitudinal analyses reported in the literature of cortical thickness data to assess disease progression in neurodegenerative conditions^19–21^, this has not yet been assessed through SCNs. In our study, despite the absence of cortical atrophy in patients, we observed features of increased network connectivity and network integration at baseline among those patients who later converted to MS. This was followed by a clear trend towards an overall loss of network connectivity over time and a deterioration of the network’s ability to integrate information, which was statistically significant in those patients who converted to MS during the follow-up. None of these reorganisational changes, either at baseline or during follow-up, were observed in non-converters’ or HCs’ networks. These results suggest that SCN analyses may be more sensitive to pathological processes occurring at these very early stages of MS than conventional analyses of cortical thickness data. Of note, the experimental groups of our study were biased towards females, indicating they were representative of the population of MS, which mainly affects females, especially with the relapse-onset phenotypes. On the other hand, in our analyses, we had initially adjusted our cortical thickness data for age and gender. This step was performed to ensure that the potential confounding effect of gender in our results was minimised or even eliminated.

Importantly, these results are in line with the idea that SCN analysis provides complementary information to more classical analyses of structural damage because they account for aspects of the data that conventionally were considered, at most, as nuisance parameters. In SCN analysis, instead, such correlations are no longer nuisance parameters to be allowed for when computing the uncertainty with which a given parameter of interest is estimated, but they are the main parameters of interest.

Disruption of SCNs was first described in MS in 2009 by He *et al*.^22^, and in 2014, Tewarie *et al*.^11^, found that SCNs disruption in MS was echoed by changes in functional networks obtained through magnetoencephalography. However, the cross-sectional design of those studies did not allow the characterisation of the dynamics of such disruption, as opposed to our study, where we could observe that the disruption occurred after an initial *explosion* of SCN connectivity, suggesting the presence of a network reorganisation after an initial perturbation, which would have been overlooked should we not had used a longitudinal design. Importantly, our longitudinal design also allowed us to evaluate the clinical relevance of such reorganisational network changes, which would not have been possible if we had only considered a single time point. Notably, such initial *explosion* of SCN connectivity observed in patients and especially in patients who later on converted to MS was mainly at the expense of negative correlations across cortical areas, as can be observed in Fig. 5. As time went by, such strong correlations tended to disappear, especially in the MS converters’ network. At the end of the follow-up, both patients’ networks had lower connectivity and global and local efficiency than the HCs’ network (although non-significantly) (Supplementary Tables 6–9).

**Figure 4:**
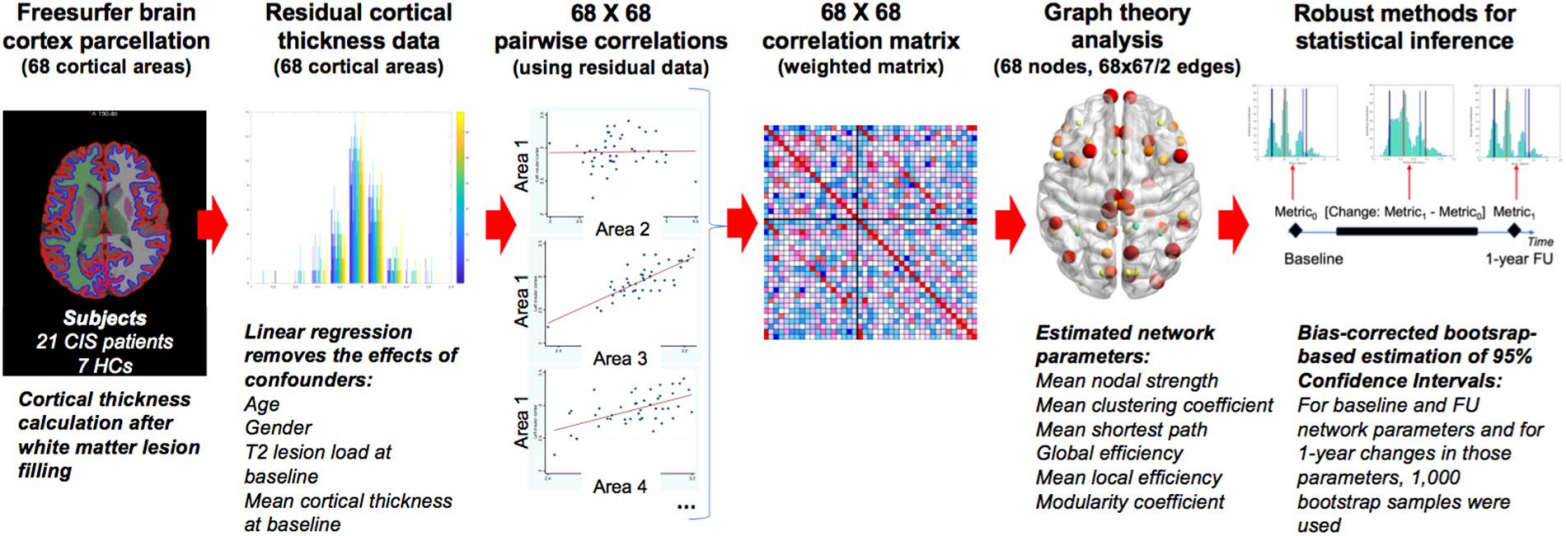
Study pipeline. This is the study pipeline that we followed to obtain the network parameters at each time point and also the changes over 1 year. For each parameter and time point, and for each parameter change, 1000 bootstrap samples were used to compute the 95% Confidence Intervals. See main text for more details. *Abbreviations:* CIS: clinically isolated syndrome; HCs: healthy controls; FU: follow-up.

Structural covariance networks are built upon the assumption that, in the healthy brain, two brain cortical areas showing morphological similarity in terms of their cortical thickness are physically and/or functionally connected^5,8,23,24^. However, in the diseased brain, the interpretation of modified or disrupted covariance networks is not straightforward. The time scale for the shaping of the covariance networks in the healthy brain is believed to span many years^7^, and it is therefore unlikely that their abnormal configuration or disruption, especially if observed over the short time period of one year, can be attributed to a disease-related damage in the underlying physical or functional connection between cortical areas. Besides, the fact that our cortical networks were built using cortical thickness values adjusted for lesion load (and other confounders) makes it unlikely that the disruption of WM tracts is the cause of the alteration in the correlations between cortical areas. On the other hand, it is also unlikely that the initially increased covariance network connectivity observed in those patients who later on converted to MS and its subsequent disruption over time are unrelated events. This statement would be supported by the presence of strikingly similar values of SCN connectivity at one-year follow-up in converters and non-converters. We speculate that these early reorganisational changes could reflect the presence of a *microscopic* inflammatory cortical process around the time of the CIS in those who will experience early future conversion, leading to an initially more homogeneous cortex among future MS converters manifesting as greater connectivity. Importantly, all cortical thickness measures used to build SCNs had been adjusted for white matter lesion load at baseline, implying that any presence of cortical inflammation at the time of the CIS must have behaved independently of macroscopic white matter lesion load. However, since we used 1.5 T scans, we could not assess the presence of cortical lesions in our patients^25^, so we could not explore this hypothesis further. Yet we did explore, in a post-hoc analysis, the effect of the lesion load on the actual network metrics (Supplementary Tables 10–13). This analysis showed that the metrics of the HCs’ network were almost unchanged, as expected, when using cortical thickness unadjusted for lesion load, though adjusted for age, gender and mean cortical thickness (see Methods for full details), as compared with the ones obtained with fully adjusted cortical thickness values. Instead, the metrics of the patients’ networks, especially the networks for all CIS patients and clinical MS converters, tended to show, in general, lower connectivity than the corresponding *adjusted* networks. This was especially evident for the 12-month follow-up networks. Therefore, this implied that, in general, differences between patients’ and controls’ networks or between converters’ and non-converters’ networks increased, as compared to *fully adjusted* cortical networks. This supports the idea that the *explosion* of connectivity at onset was indeed probably associated with greater inflammation. Future studies with high-field multi-modal MR images are warranted to understand the pathological substrate underlying cortical network changes in early MS.

When patients were divided into two groups – those who *clinically* converted and those who did not convert to MS within the one-year follow-up, SCN analysis revealed striking differences between groups: at baseline, converters’ network showed signs of greater connectivity than non-converters’ network. However, during the follow-up, whereas MS converters exhibited longitudinal changes consistent with progressive disruption of SCNs, the non-converters’ network overall showed minimal, non-significant, changes over time, mirroring the behaviour of HCs’ network. This is the first time that clinical relevance of covariance cortical networks in MS has been reported from longitudinal data. Reassuringly, similar results were found when MS converters were compared to non-MS converters according to the 2010 McDonald criteria^18^ (Supplementary Tables 4 and 5).

Importantly, the similarity between all patients’ and MS converters’ networks was also reflected in the regional distribution of nodal connectivity changes, reinforcing the concept that SCN changes may be clinically relevant: losses of nodal strength, clustering coefficient and local efficiency seemed to be more obvious in pre- and post-central gyri and the paracentral lobule, bilaterally, not only in (all) patients’ network, but also in the MS converters’ network, as shown in Fig. 3. Interestingly, these central regions, which in this context would indicate those with greatest loss of similarity with other cortical regions, have been also identified, in diffusion-based networks, as *hubs* and *rich-club members*^26^. *Rich-club members* are nodes with a high number of anatomical connections, preferentially involving other highly connected nodes, which are known to play key roles in the hierarchical organisation of the human brain^26^ and seem to possess specific microstructural features^27^.

Although SCN analyses cannot elucidate the biological processes underlying the observed changes in covariance patterns, this does not reduce the importance of our findings. Our study may have revealed a crucial aspect of MS pathology detectable in those who convert early to MS, indicating its clinical relevance, and only when an analysis of the covariance across cortical regions’ thickness is performed. Besides, although observed at the group level (because of the nature of SCNs), our results provide valid grounds for the generation of new hypotheses about unexplored mechanisms underlying disability progression. In this case, it is likely that the ultimate cause associated with such cortical network reorganisational changes is also related to conversion to MS, rather than being the presence of such SCN changes *per se* the factor actually conferring a higher risk for conversion. For instance, a post-hoc analysis comparing patients with supratentorial lesions only and patients with supra- and infratentorial lesions, at any time during the 12-month follow-up period, revealed that the network of those patients with both supra- and infratentorial lesions behaved similarly to that of patients with an early conversion to MS (Supplementary Tables 14 and 15). Whereas this reflects the known relationship between presence of infratentorial lesions and a worse prognosis, it might also reflect a possible mechanism through which the presence of infratentorial lesions entails this worse prognosis. In any case, further studies combining SCN analysis and other connectivity and structural imaging methods, such as resting-state fMRI, high-resolution magnetisation transfer imaging^28^ and diffusion-weighted imaging^29^, are required to elucidate this point. Importantly, SCN methodology can also help assess how the presence of certain well-known risk factors, such as smoking habit or low vitamin D levels, which many times can only be assessed at the group level, or how the implementation of population-level measures, such as education programmes about healthier diet or habits, can modify multiple pathological aspects of the disease, at once (i.e. in a multivariate manner) and straightforwardly.

Covariance networks are built based on correlations at the group level. This means that classical statistical methods such as t-test or regression-type approaches cannot assess differences between groups or between time points. Even if for many network metrics, in particular for those implying node-level estimations (i.e. nodal clustering coefficient or nodal local efficiency), mean values are estimated and there is a network-level variability based on the values estimated for the different nodes, this variability could not be used to carry out statistical tests based on the group variance given the strong dependence of such values within the network. In this study, we utilised a bootstrap-based approach to estimate the limits of the 95% confidence intervals for the parameters that define the networks (e.g. mean nodal strength), needed to estimate whether two networks are actually different or whether an observed change over time is statistically significant. Bootstrap-based approaches rely on the variability of the original data to estimate the confidence intervals, without making any distributional assumptions^13,30^. Thus, they are much more robust and conservative than classical inference methods, implying that the risk of type I error is actually below the nominal 5% and that the changes we observed in the parameters of the converters’ network are likely to be genuine. Of note, the use of robust bootstrapping techniques to assess the uncertainty of estimated parameters is particularly relevant when these parameters are derived from a given network, where it would not be correct to make parametric assumptions based on the distribution of individual nodal metrics within the same network to estimate the uncertainty of the network-defining parameter^30^.

Although SCN analysis challenges the possibility of drawing conclusions at the individual level, it appears as an attractive and straightforward method to assess the relationship between complex multi-dimensional predictors, such as cortical thickness data, and a given clinical outcome. Although some authors have proposed individualised covariance networks using cortical thickness data to study neurodegeneration^31,32^, these have not yet been published in MS. Besides, the relationship between individual-level and group-level network disruptions in other neurodegenerative conditions has not yet been assessed either.

A further methodological consideration relates to the fact that thickness values from each region were treated equally in the statistical analysis, as previous studies have reported^8^. This implies that we did not account for the different volumes of the brain areas, although this might not be relevant in our case, since we were interested in the associations between cortical thicknesses, which are those that are believed to reflect underlying physical or functional connections^8^. Another consideration is that we constructed the weighted graphs using the absolute values of the Pearson’s correlation coefficients of the covariance matrices, as described previously^8^. Although a visual inspection of the correlation matrices at baseline and at 12-month follow-up suggest that increased baseline connectivity in patients (and especially in early MS converters) was mainly at the expense of negative correlations, a formal connectivity analysis for positive and negative graphs was not performed in this study. Although it may be interesting to assess independently networks derived from positive and negative correlations, this was beyond the scope of this study. This analysis would also pose other challenges with methodology and interpretation and may deserve attention in future studies.

Another consideration refers to the fact that we used the 2010 revisions to the McDonald Criteria^18^ for the diagnosis of McDonald MS, instead of the most recent ones, published in 2018^33^. The 2010 revisions to the McDonald Criteria for the diagnosis of MS were the current criteria for the diagnosis of MS when this study was designed, in 2015. We acknowledge that the results might be slightly different from those that we present now if we had used the 2017 revisions of the McDonald criteria.

Finally, the small sample size, especially in controls, is an important limitation of the study. Although the lack of significant changes in controls could have theoretically been attributed to controls’ smaller sample size, when we compared MS converters with non-converters, where also covariance networks were built with small sample sizes, MS converters showed statistically significant changes that were not observed in non-converters. Importantly, because all our inferences related to SCNs were made through robust bootstrap-based approaches, it is possible that the observed significant results are in fact genuine. However, it is also true that the sample size was low even for using a bootstrap approach, therefore the robustness of the network of anatomical covariance analysis was not guaranteed, and this could have biased the statistical results. We therefore consider these results as preliminary and acknowledge the need to replicate them in larger cohorts.

In conclusion, SCNs of patients with their first inflammatory-demyelinating episode of the CNS, and especially of those who convert to MS, show clear reorganisational changes in the absence of detectable cortical atrophy. Thus, covariance network analysis not only is more sensitive than conventional analysis of structural data but also seems to unveil clinically relevant aspects of this condition. Of note, the low sample size indicates this is preliminary work and the results and conclusions derived from it should be taken with caution and be confirmed in larger cohorts. In any case, the fact that covariance network analysis can be applied retrospectively to large cohorts of patients who have undergone T1-weighted scans within other protocols makes this technique a powerful and straightforward method to help us understand pathological processes underlying disease progression in MS.

## Methods

### Subjects

We included consecutive patients with a diagnosis of optic neuritis as their first demyelinating attack of the central nervous system (i.e. CIS) within the previous four weeks. All patients underwent clinical assessments, at baseline, 3, 6 and 12 months. At each visit, apart from visual assessments, we recorded whether the patient had suffered a second clinical attack. Additionally, we also recorded the presence of new white matter T2 lesions and whether these occurred in different locations, fulfilling dissemination in time and space criteria and therefore fulfilling the diagnostic criteria of MS according to the 2010 revisions to the McDonald criteria^18^ (Table 1). This has been reported elsewhere^16,17^. We also included a group of age-matched healthy controls (HCs). All participants underwent MRI scans. The study was approved by the Ethics Committee of the National Hospital for Neurology and Neurosurgery, part of the University College London Hospitals NHS Foundation Trust. This is an observational study and all methods were performed in accordance with the relevant guidelines and regulations. All participants provided informed written consent.

### MRI analysis

All MRI scans were performed with a 1.5 T GE Signa Echospeed MRI (Milwaukee, WI) scanner. The scanner maximum gradient strength was 33 mT m^−1^.

#### Acquisition of brain structural scans

For all subjects, we acquired the following images at baseline, 3, 6 and 12 months: axial oblique, proton-density (PD), dual echo, fast spin echo images (resolution: 0.9 × 0.9 × 5 mm^3^), as previously described^14–17^; axial three-dimensional fast prepared spoiled gradient recall (3D-FSPGR, 3D T1-weighted) (resolution: 1.2 × 1.2 × 1.2 mm^3^).

T2 hyperintense WM lesions were manually outlined by an experienced observer from the PD-weighted images using the semi-automated edge finding tool from JIM (JIM v6.0, Xinapse systems, Aldwincle, UK, http://www.xinapse.com). PD-weighted lesion masks were co-registered to the 3D-T1 images using a pseudo-T1 image generated by subtracting the PD from the T2-weighted image^34^. Lesion masks were transformed from native space to 3DT1 space. The 3DT1 images were filled using a non-local patch match lesion filling technique^35^.

#### Measurement of cortical thickness

We calculated cortical thicknesses for 68 bilateral brain cortical areas using the FreeSurfer version 5.3 longitudinal stream^36^, the technical details of which have been explained elsewhere^36–38^. Briefly, this included skull-stripping, intensity normalisation, non-linear registration to Talairach space, segmentation, estimation of brain surfaces, and surface parcellation. We visually assessed the final segmentation, and re-ran the pipeline after manual correction in cases of incorrect surface estimation. To perform unbiased longitudinal image analysis, we created a symmetric within-subject template^39^. Next, all the steps were re-initialised for each time point using the common information to increase reliability and statistical power^36^. We extracted cortical thickness values for each cortical parcellation according to Desikan-Killiany atlas^38^.

#### Structural covariance network analysis

**Figure 5:**
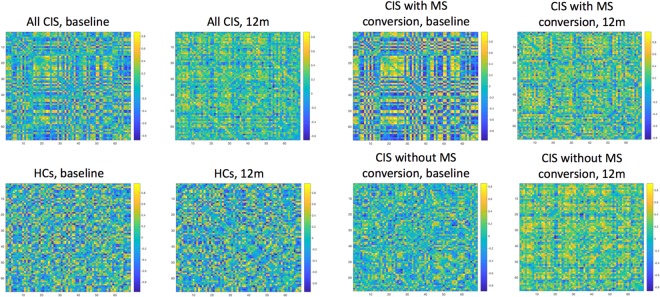
Correlation matrices. This figure shows the correlation matrices for each one of the groups (all CIS, HCs, CIS with early conversion, CIS without early clinical conversion), at baseline and at follow-up. From these correlation matrices, we extracted the absolute values of the correlations and built the weighted graphs, used to obtain network metrics. *Abbreviations:* CIS: clinically isolated syndrome; HCs: healthy controls; MS: multiple sclerosis.

Construction of weighted structural covariance networks (Fig. 5): We constructed two SCNs, one at baseline and one at 12-month follow-up, for each of these four groups – a) HCs, b) all CIS patients, c) MS-converter CIS patients and d) non-converter CIS patients, as follows (of note, although SCNs were also built at 3- and 6-month follow-up, the main analysis involved only baseline and 12-month follow-up SCNs):

1. In 3 patients who did not attend the 12-month follow-up but who attended the 0, 3 and 6-month time points, cortical thickness at 12-month follow-up was imputed using each patient’s cortical thickness trajectory, i.e. a single imputation technique based on simple linear regression.
2. Because we were interested in knowing the relationships between cortical thickness values across regions whilst adjusting for the effects of possible confounders, we removed variability in the cortical thickness data related to lesion load, cortical atrophy, age or gender, all cortical thickness values, for all subjects and time points, regressing all cortical thickness values at once over baseline lesion load and baseline cortical thickness, age and gender. Total intracranial volume (TIV) obtained through GIF segmentation^40^ of the T1-weighted images was also explored as a covariate. Yet, given its potential association with age and gender, it was only kept if significant at 5% level, to avoid collinearity. The residuals of these regression models were considered the new, *adjusted*, cortical thickness values to be used in subsequent steps^8^. For this step, controls were assigned a lesion load equal to zero mL, as previously performed^17,41,42^, to be able to obtain the residuals for controls through the same regression model as that used to obtain patients’ residuals. This allowed us to maintain the original relationships between patients and controls. The assumptions of the linear regression were checked through the analysis of the residuals.
3. Pairwise correlation matrices using *adjusted* cortical thickness values, with Pearson’s correlation coefficient as the association measure, were obtained for each group (i.e. CIS patients, HCs, CIS patients with early conversion to MS and CIS patients without early conversion to MS), at baseline and at 12-month follow-up, using MATLAB (The MathWorks, Inc., Natick, Massachusetts, USA) (Fig. 4).
4. Since we aimed to build weighted networks reflecting the strength of the correlation regardless of whether there was a positive or negative association^8^, we obtained correlation matrices with the absolute value of Pearson’s correlation coefficients.

Obtaining network topological metrics: For each weighted matrix, considered as the numerical representation of a network with 68 nodes (i.e. 68 cortical areas) and edges indicating the strength of the connection between two cortical areas, we obtained the following network topological metrics at the network level. That is, for each metric, we obtained a value for each network (i.e. for each group and time point). We used the freely available Brain Connectivity Toolbox^43^ (https://sites.google.com/site/bctnet/) in MATLAB. The metrics were divided into three groups:

1. *Metrics of nodal connectivity*: *mean nodal strength and mean clustering coefficient*
  These metrics describe the connectivity of each node in the network. Mean nodal strength is the average, across all nodes, of the nodal strength, defined as the sum of the correlation coefficients of the edges emerging from a given node. Thus, a node with a high strength indicates that the node is very well connected, and a network with high mean nodal strength indicates a very well-connected network. Importantly, in this context, highly connected networks indicate a high degree of similarity across cortical regions.
  Nodal clustering coefficient reflects the connectivity among the neighbours of a given node, and can be understood as the probability that each pair of nodes that are connected to a given node are also connected among themselves. It can be defined as in [1]:
  1 $${\rm{Clustering}}\,{\rm{coefficient}}\,{\rm{of}}\,{\rm{node}}\,i=\frac{Number\,of\,pairs\,of\,node\,i\mbox{'}\,s\,neighbours\,that\,are\,connected\,}{Number\,of\,pairs\,of\,node\,i\mbox{'}\,s\,neighbours}$$
  Therefore, the mean clustering coefficient is the average clustering coefficient across all nodes of the network^44^. In this context, a network with a high mean clustering coefficient would indicate that cortical regions have, in general, strong similarities in terms of cortical thickness with neighbour regions.
2. *Metrics of network integration: mean shortest path, global efficiency and local efficiency*
  These metrics describe the ability of the network to efficiently integrate information across nodes. Smaller values of mean shortest path infer more efficient information transfer between nodes and greater information integration within the network^45^. Mean shortest path (or *characteristic path length, L*) is the average of the shortest path lengths between all pairs of nodes in the network^43^, and can be defined as in [2]:
  2 $${\rm{L}}=\frac{1}{N}\sum _{i}{l}_{i}=\frac{1}{N(N-1)}\,\sum _{i\ne j}{l}_{ij},$$
  where *N* is number of nodes in the network; *l*_*i*_ is the average shortest path from node i to all other nodes; and *l*_*ij*_ is the shortest path length from node *i* to node *j*.
  Global efficiency (*E*_*glob*_) is the reciprocal of the harmonic mean of the shortest path lengths of the entire network^45^, and is defined as in [3]:
  3 $${E}_{glob}=\frac{1}{L^{\prime} }=\frac{1}{N(N-1)}\sum _{i\ne j}\frac{1}{{l}_{ij}},$$
  where *L*′ is the harmonic mean of the mean shortest path; *N* is number of nodes in the network; and *l*_*ij*_ is the shortest path length from node *i* to node *j*.
  Local efficiency, which is a node-level metric of integration of information, is the reciprocal of the harmonic mean of the shortest path lengths of the *subgraph* (i.e. *subnetwork*) defined by the neighbours of a given node^45^. Local efficiency of node i (E_loc_ (i)), is defined as in [4]:
  4 $${E}_{loc}(i)=\frac{1}{{N}_{{G}_{i}}({N}_{{G}_{i}}-1)}\,\sum _{j,h\in {G}_{i}}\frac{1}{{l}_{jh}},$$
  where *G*_*i*_ is the subnetwork including all nodes that are neighbours of node *i*; ${N}_{{G}_{i}}$is the number of nodes belonging to subnetwork *G*_*i*_; and *l*_*jh*_ is the shortest path length from node *j* to node *h* belonging to subnetwork *G*_*i*_.
  Higher global and local efficiency values indicate greater ability to integrate information, globally and locally, respectively. Mean local efficiency is the across-node average of local efficiency values of the nodes of the network^43^.
3. *Metric of network organisation: modularity coefficient*
  Modularity coefficient (ModC) assesses how groups of nodes are clustered in local groups. That is, ModC, describes how well a network can be subdivided into groups of nodes (i.e. modules), highly correlated among each other, creating small communities inside the network.

### Statistical analysis

#### Descriptive and structural imaging data analyses

Descriptive statistics are reported as mean (standard deviation [SD]) or median (range), depending on the nature of the variable.

We assessed changes in mean cortical thickness over one year as part of the preliminary analysis using mixed-effects models, where ‘mean cortical thickness’ was the dependent variable and ‘time’, a ‘binary group indicator’ (i.e. patient/control) and an interaction term such as ‘time X group indicator’ were the main explanatory variables. Whenever the interaction term was significant, it was assumed that the rate of change in cortical thickness differed between the two groups. All models were adjusted for age, gender and lesion load at baseline (HCs were assigned lesion load equal to zero). For the final analyses, total intracranial volume was not included among the covariates: it was not significant in any of the models and its introduction did not change the direction or size of the effects of the other covariates. Mixed-effects models allowed for repeated measures within the same subject^17,46^ and their random structure consisted of a random intercept (for each subject) and random slope (for time, which was nested in ‘subject’). Whenever residuals from mixed-effects regression models showed deviation from normality, 95% confidence intervals (95% CI) and p-values were obtained using bias-corrected non-parametric bootstrap with 1000 replicates.

All these statistical analyses were carried out with Stata 14.1.

#### SCN analysis in all CIS patients versus healthy controls

Since all network parameters were obtained at the network level, i.e. we had a value for each network, classical statistical approaches could not be used to compute the 95% confidence intervals (95% CI) of the metric values at baseline and their changes over time, so we employed a bootstrap-based approach with these steps:

1. For each network metric, we initially obtained our baseline value and 12-month follow-up value. From the subtraction of these two, we obtained the change in that network. These were our *original estimates* for *baseline*, 12-month *follow-up* and *change* values.
2. For the groups of CIS patients and HCs, we then created 1000 *bootstrap samples* (i.e. random samples of *n* subjects chosen –allowing sampling with replacement– from each group, being *n* = sample size of the original group).
3. In each bootstrap sample, covariance matrices for baseline and follow-up values were computed using exactly the same methodology as the one used with the original group.
4. From each covariance matrix, network topological measures were computed, for baseline and 12-month follow-up. The difference between baseline and follow-up metrics was also obtained for each bootstrap sample. Importantly, for this step (i.e. to compute the difference between baseline and follow-up), the same subset of bootstrap samples (subset of subjects) was used. These were our *bootstrap estimates* for *baseline*, 12-month *follow-up* and *change* values.
5. Once all bootstrap samples were analysed, distributions of *bootstrap estimates* were obtained. These were then compared against the original estimates and the *bootstrap-based* 95% CI were obtained for each metric and each group, at baseline and follow-up. *Bootstrap-based* 95% CI were also obtained for each *change* in the network metric. For the estimation of the *change*, whenever the 95% CI did not include the null value (=0), we assumed the change was significant over time. To compute differences between two groups, a 1000 × 1000 matrix was obtained, where all possible *combinations of differences* between the bootstrap estimates (for *baseline*, *follow-up* or *change* parameters) for the two groups were calculated. Original differences between the two groups that we wanted to compare were then plotted against such 1,000,000-item distribution of combinations and *combined-bootstrap-based* 95% CI were obtained for these original differences. Whenever these *combined-bootstrap-based* 95% CIs did not include the null value (=0), we inferred significant differences between the two groups for a particular *baseline*, *follow-up* or *change* parameter. All these analyses were carried out with MATLAB.

#### SCN analysis in CIS patients who convert versus those who do not convert to MS during follow up

The same steps described above were also applied to assess the 95% CI of baseline, follow-up and change values for the network metrics in the two groups of CIS patients with and without early conversion to MS. Different behaviours between the two groups in a given network parameter were interpreted as this metric being potentially relevant from a clinical point of view.

A post-hoc analysis comparing the networks of patients who were diagnosed with McDonald MS (i.e. either they had a second attack or fulfilled the criteria for dissemination in time and space during the follow-up) was also performed (results shown in Supplementary Tables 4 and 5).

### Data availability

The datasets generated during and/or analysed during the current study are available from the corresponding author on reasonable request.

## Electronic supplementary material

Supplementary Tables and Figures

## References

[1] Fisniku LK (2008). Gray matter atrophy is related to long-term disability in multiple sclerosis. Ann Neurol.

[2] Geurts JJ, Barkhof F (2008). Grey matter pathology in multiple sclerosis. Lancet Neurol.

[3] Eshaghi, A. *et al*. Data-driven staging of atrophy progression in multiple sclerosis. *Brain (under review)* (2017).

[4] Filippi M (2013). Assessment of system dysfunction in the brain through MRI-based connectomics. Lancet Neurol.

[5] Alexander-Bloch A, Giedd JN, Bullmore E (2013). Imaging structural co-variance between human brain regions. Nat Rev Neurosci.

[6] Clayden JD (2013). Imaging connectivity: MRI and the structural networks of the brain. Funct Neurol.

[7] Evans AC (2013). Networks of anatomical covariance. Neuroimage.

[8] Lerch JP (2006). Mapping anatomical correlations across cerebral cortex (MACACC) using cortical thickness from MRI. Neuroimage.

[9] Charil A (2007). Focal cortical atrophy in multiple sclerosis: relation to lesion load and disability. Neuroimage.

[10] He Y, Chen ZJ, Evans AC (2007). Small-world anatomical networks in the human brain revealed by cortical thickness from MRI. Cereb Cortex.

[11] Tewarie P (2014). Disruption of structural and functional networks in long-standing multiple sclerosis. Hum Brain Mapp.

[12] Sanchez-Catasus CA (2017). Subtle alterations in cerebrovascular reactivity in mild cognitive impairment detected by graph theoretical analysis and not by the standard approach. Neuroimage Clin.

[13] Melie-Garcia, L. *et al*. Networks of myelin covariance. *Hum Brain Mapp*, 10.1002/hbm.23929 (2017). 10.1002/hbm.23929 PMC5873432 29271053

[14] Jenkins T (2010). Dissecting structure-function interactions in acute optic neuritis to investigate neuroplasticity. Hum Brain Mapp.

[15] Jenkins TM (2011). Early pericalcarine atrophy in acute optic neuritis is associated with conversion to multiple sclerosis. J Neurol Neurosurg Psychiatry.

[16] Jenkins TM (2010). Neuroplasticity predicts outcome of optic neuritis independent of tissue damage. Ann Neurol.

[17] Tur C (2016). Longitudinal evidence for anterograde trans-synaptic degeneration after optic neuritis. Brain.

[18] Polman CH (2011). Diagnostic criteria for multiple sclerosis: 2010 revisions to the McDonald criteria. Ann Neurol.

[19] Mak E (2015). Baseline and longitudinal grey matter changes in newly diagnosed Parkinson’s disease: ICICLE-PD study. Brain.

[20] Weston PS (2016). Presymptomatic cortical thinning in familial Alzheimer disease: A longitudinal MRI study. Neurology.

[21] Duering M (2015). Acute infarcts cause focal thinning in remote cortex via degeneration of connecting fiber tracts. Neurology.

[22] He Y (2009). Impaired small-world efficiency in structural cortical networks in multiple sclerosis associated with white matter lesion load. Brain.

[23] Gong G, He Y, Chen ZJ, Evans AC (2012). Convergence and divergence of thickness correlations with diffusion connections across the human cerebral cortex. Neuroimage.

[24] Hunt BA (2016). Relationships between cortical myeloarchitecture and electrophysiological networks. Proc Natl Acad Sci USA.

[25] Arrambide G (2017). Lesion topographies in multiple sclerosis diagnosis: A reappraisal. Neurology.

[26] van den Heuvel MP, Sporns O (2011). Rich-club organization of the human connectome. J Neurosci.

[27] Ooi, J. *et al*. In *ECTRIMS (Poster presentation)* (Paris, 25–28 October 2017, 2017).

[28] Samson RS (2014). Investigation of outer cortical magnetisation transfer ratio abnormalities in multiple sclerosis clinical subgroups. Mult Scler.

[29] Grussu F (2017). Neurite dispersion: a new marker of multiple sclerosis spinal cord pathology?. Ann Clin Transl Neurol.

[30] Gel YR, Lyubchich V, Ramirez Ramirez LL (2017). Bootstrap quantification of estimation uncertainties in network degree distributions. Sci Rep.

[31] Tijms BM (2013). Single-subject grey matter graphs in Alzheimer’s disease. PLoS One.

[32] Tijms BM (2015). Grey matter networks in people at increased familial risk for schizophrenia. Schizophr Res.

[33] Thompson AJ (2018). Diagnosis of multiple sclerosis: 2017 revisions of the McDonald criteria. Lancet Neurol.

[34] Hickman SI, Barker GJ, Molyneux PD, Miller DH (2002). Technical note: the comparison of hypointense lesions from ‘pseudo-T1’ and T1-weighted images in secondary progressive multiple sclerosis. Mult Scler.

[35] Prados F (2016). A multi-time-point modality-agnostic patch-based method for lesion filling in multiple sclerosis. Neuroimage.

[36] Reuter M, Schmansky NJ, Rosas HD, Fischl B (2012). Within-subject template estimation for unbiased longitudinal image analysis. Neuroimage.

[37] Dale AM, Fischl B, Sereno MI (1999). Cortical surface-based analysis. I. Segmentation and surface reconstruction. Neuroimage.

[38] Desikan RS (2006). An automated labeling system for subdividing the human cerebral cortex on MRI scans into gyral based regions of interest. Neuroimage.

[39] Reuter M, Rosas HD, Fischl B (2010). Highly accurate inverse consistent registration: a robust approach. Neuroimage.

[40] Cardoso MJ (2015). Geodesic Information Flows: Spatially-Variant Graphs and Their Application to Segmentation and Fusion. IEEE Trans Med Imaging.

[41] Khaleeli Z (2008). Magnetization transfer ratio in gray matter: a potential surrogate marker for progression in early primary progressive multiple sclerosis. Arch Neurol.

[42] Tur C (2014). HLA-DRB1*15 influences the development of brain tissue damage in early PPMS. Neurology.

[43] Rubinov M, Sporns O (2010). Complex network measures of brain connectivity: uses and interpretations. Neuroimage.

[44] Watts DJ, Strogatz SH (1998). Collective dynamics of ‘small-world’ networks. Nature.

[45] Latora V, Marchiori M (2001). Efficient behavior of small-world networks. Phys Rev Lett.

[46] Petrova, N., Carassiti, D., Altmann, D. R., Baker, D. & Schmierer, K. Axonal loss in the multiple sclerosis spinal cord revisited. *Brain Pathol*, 10.1111/bpa.12516 (2017). 10.1111/bpa.12516 PMC8028682 28401686

