# Supplementary material for: Structural cortical network reorganization associated with early conversion to multiple sclerosis
Source: Sci Rep. 2018 Jul 16;8:10715. doi: 10.1038/s41598-018-29017-1 (PMC6048099; doi:10.1038/s41598-018-29017-1)
Supplement: Supplementary file 1 — Supplementary Tables and Figures [file 41598_2018_29017_MOESM1_ESM.pdf]

**Title: Structural cortical network reorganization associated with early conversion to multiple sclerosis**

**Authors:** C. Tur, A. Eshaghi, D.R. Altmann, T.M. Jenkins, F. Prados, F. Grussu, T. Charalambous, A. Schmidt, S. Ourselin, J.D. Clayden, C.A.M.G. Wheeler-Kingshott, A.J. Thompson, O. Ciccarelli, A.T. Toosy

**Corresponding author:** Carmen Tur, MD, MSc, PhD; Queen Square MS Centre, Queen Square MS Centre, Department of Neuroinflammation, UCL Institute of Neurology, University College of London (UCL), London WC1B 5EH, UK; tel: +44 203 1087446; e-mail: c.tur@ucl.ac.uk.

## SUPPLEMENTARY TABLES

### Supplementary Table 1. Lesion locations

| | All CIS | CIS-CIS[a] | CIS-MS[b] | CIS-CIS[a] vs. CIS-MS[b], p-value[c] |
|---|---|---|---|---|
| N finally included | 21 | 12 | 9 | - |
| N patients with supratentorial lesions | | | | |
| Baseline | 16 | 7 | 9 | 0.027 |
| 3 months | 16 | 7 | 9 | 0.027 |
| 6 months | 15 | 6 | 9 | 0.020 |
| 12 months | 15 | 7 | 8 | 0.090 |
| N patients with infratentorial lesions | | | | |
| Baseline | 7 | 3 | 4 | 0.350 |
| 3 months | 7 | 3 | 4 | 0.350 |
| 6 months | 6 | 2 | 4 | 0.202 |
| 12 months | 7 | 4 | 3 | 0.914 |
| N patients with supratentorial and infratentorial lesions | | | | |
| Baseline | 7 | 3 | 4 | 0.350 |
| 3 months | 7 | 3 | 4 | 0.350 |
| 6 months | 6 | 2 | 4 | 0.202 |
| 12 months | 7 | 4 | 3 | 0.914 |

**Supplementary Table 1 (legend). a:** patients who did not have a second attack during the follow-up; **b:** patients who had a second attack during the follow-up; **c:** chi-square t-test.

## Supplementary Table 2. Estimated baseline and follow-up values of network parameters, in all CIS patients and controls

| | CIS patients (all) | HCs | Patients vs. HCs, estimated p-value |
|---|---|---|---|
| *1. Measures of nodal connectivity* | | | |
| **Mean nodal strength, estimated value (bootstrap-based 95% CI)** | | | |
| Baseline | 20.11 (13.98 to 30.59) | 24.08 (18.69 to 37.13) | 0.60 |
| Follow-up | 16.49 (13.13 to 22.58) | 23.50 (18.17 to 37.00) | 0.25 |
| **Mean clustering coefficient, estimated value (bootstrap-based 95% CI)** | | | |
| Baseline | 0.30 (0.22 to 0.45) | 0.31 (0.23 to 0.50) | 0.99 |
| Follow-up | 0.23 (0.19 to 0.31) | 0.30 (0.22 to 0.50) | 0.30 |
| *2. Measures of nodal distance* | | | |
| **Mean shortest path, estimated value (bootstrap-based 95% CI)** | | | |
| Baseline | 3.11 (2.49 to 3.73) | 2.46 (2.00 to 2.73) | 0.10 |
| Follow-up | 3.47 (2.99 to 3.92) | 2.49 (2.02 to 2.76) | **<0.001** |
| **Global efficiency, estimated value (bootstrap-based 95% CI)** | | | |
| Baseline | 0.41 (0.35 to 0.53) | 0.45 (0.39 to 0.62) | 0.60 |
| Follow-up | 0.36 (0.31 to 0.42) | 0.45 (0.39 to 0.62) | 0.08 |
| **Mean local efficiency, estimated value (bootstrap-based 95% CI)** | | | |
| Baseline | 0.33 (0.25 to 0.47) | 0.34 (0.27 to 0.53) | 0.90 |
| Follow-up | 0.27 (0.22 to 0.34) | 0.34 (0.27 to 0.53) | 0.25 |
| *3. Measures of network organisation* | | | |
| **Modularity coefficient (bootstrap-based 95% CI)** | | | |
| Baseline | 0.07 (0.03 to 0.10) | 0.10 (0.08 to 0.13) | 0.10 |
| Follow-up | 0.09 (0.07 to 0.11) | 0.11 (0.09 to 0.14) | 0.30 |

**Supplementary Table 2 (footnote).** Comparison between CIS patients and HCs. See main text for full details on the methods used. Significant p-values are indicated with bold letters. *Abbreviations:* CI: confidence interval; CIS: clinically isolated syndrome; FU: follow-up; HCs: healthy controls;

**Supplementary Table 3. Estimated baseline and follow-up values of network parameters in patients with and without clinical MS conversion**

| | Patients with clinical MS conversion | Patients without clinical MS conversion | Patients with vs. without MS conversion, estimated p-value |
|---|---|---|---|
| *1. Measures of nodal connectivity* | | | |
| **Mean nodal strength, estimated value (bootstrap-based 95% CI)** | | | |
| Baseline | 30.96 (21.13 to 42.96) | 17.23 (13.98 to 23.02) | **0.02** |
| Follow-up | 20.78 (16.41 to 30.08) | 19.62 (15.22 to 26.99) | 0.80 |
| **Mean clustering coefficient, estimated value (bootstrap-based 95% CI)** | | | |
| Baseline | 0.42 (0.28 to 0.62) | 0.23 (0.19 to 0.30) | **0.02** |
| Follow-up | 0.26 (0.20 to 0.40) | 0.26 (0.21 to 0.36) | 0.99 |
| *2. Measures of nodal distance* | | | |
| **Mean shortest path, estimated value (bootstrap-based 95% CI)** | | | |
| Baseline | 2.20 (1.76 to 2.66) | 3.19 (2.82 to 3.50) | **<0.001** |
| Follow-up | 2.76 (2.34 to 3.07) | 2.91 (2.51 to 3.26) | 0.60 |
| **Global efficiency, estimated value (bootstrap-based 95% CI)** | | | |
| Baseline | 0.53 (0.42 to 0.68) | 0.38 (0.34 to 0.45) | **0.02** |
| Follow-up | 0.40 (0.35 to 0.52) | 0.41 (0.36 to 0.50) | 0.90 |
| **Mean local efficiency, estimated value (bootstrap-based 95% CI)** | | | |
| Baseline | 0.45 (0.31 to 0.63) | 0.27 (0.23 to 0.34) | **0.02** |
| Follow-up | 0.30 (0.24 to 0.43) | 0.30 (0.25 to 0.40) | 0.99 |
| *3. Measures of network organisation* | | | |
| **Modularity coefficient (bootstrap-based 95% CI)** | | | |
| Baseline | 0.06 (0.02 to 0.12) | 0.10 (0.08 to 0.12) | 0.40 |
| Follow-up | 0.09 (0.07 to 0.12) | 0.10 (0.08 to 0.12) | 0.60 |

**Supplementary Table 3 (footnote).** Comparison between patients with and without clinical MS conversion. See main text for full details on the methods used. Significant p-values are indicated with bold letters. *Abbreviations:* CI: confidence interval; CIS: clinically isolated syndrome; FU: follow-up; HCs: healthy controls;

## Supplementary Table 4. Estimated baseline and follow-up values of network parameters in patients with and without McDonald MS conversion

| | Patients with McDonald MS conversion | Patients without McDonald MS conversion | Patients with vs. without McDonald MS conversion, estimated p-value |
|---|---|---|---|
| ***1. Measures of nodal connectivity*** | | | |
| **Mean nodal strength, estimated value (bootstrap-based 95% CI)** | | | |
| Baseline | 26.9067 (18.3856 to 37.7224) | 20.5751 (16.2493 to 28.8888) | 0.40 |
| Follow-up | 17.1404 (13.8737 to 22.8255) | 22.2895 (17.5633 to 31.4500) | 0.20 |
| **Mean clustering coefficient, estimated value (bootstrap-based 95% CI)** | | | |
| Baseline | 0.3643 (0.2486 to 0.5285) | 0.2647 ( 0.2115 to 0.3775) | 0.30 |
| Follow-up | 0.2327 (0.1958 to 0.3020) | 0.2809 (0.2211 to 0.411) | 0.30 |
| ***2. Measures of nodal distance*** | | | |
| **Mean shortest path, estimated value (bootstrap-based 95% CI)** | | | |
| Baseline | 2.4610 (2.0125 to 2.9670) | 2.7878 ( 2.4021 to 3.119) | 0.40 |
| Follow-up | 3.2090 (2.8289 to 3.5262) | 2.6197 (2.2229 to 2.8963) | **0.03** |
| **Global efficiency, estimated value (bootstrap-based 95% CI)** | | | |
| Baseline | 0.4729 (0.3868 to 0.6026) | 0.4087 (0.3561 to 0.5129) | 0.40 |
| Follow-up | 0.3818 (0.3423 to 0.4462) | 0.4313 (0.3769 to 0.5441) | 0.25 |
| **Mean local efficiency, estimated value (bootstrap-based 95% CI)** | | | |
| Baseline | 0.3918 (0.2840 to 0.5453) | 0.3021 (0.2490 to 0.4118) | 0.30 |
| Follow-up | 0.2711 (0.2337 to 0.3393) | 0.3198 (0.2615 to 0.4445) | 0.30 |
| ***3. Measures of network organisation*** | | | |
| **Modularity coefficient (bootstrap-based 95% CI)** | | | |
| Baseline | 0.0655 (0.0250 to 0.1090) | 0.1022 (0.0847 to 0.1246) | 0.20 |
| Follow-up | 0.0995 (0.0820 to 0.1200) | 0.1174 (0.0984 to 0.1456) | 0.25 |

**Supplementary Table 4 (footnote).** Comparison between patients with and without McDonald MS conversion. See main text for full details on the methods used. Significant p-values are indicated with bold letters. *Abbreviations:* CI: confidence interval; CIS: clinically isolated syndrome; FU: follow-up; HCs: healthy controls.

**Supplementary Table 5. One-year changes in SCN parameters, in CIS patients with and without McDonald MS conversion**

| | Patients with McDonald MS conversion | Patients without McDonald MS conversion | Patients with vs. without McDonald MS conversion, estimated p-value |
|---|---|---|---|
| *1. Measures of nodal connectivity* | | | |
| **Mean nodal strength, estimated value (bootstrap-based 95% CI), p-value** | | | |
| Change from baseline to 1 year | -9.7663 (-18.7913 to -1.7113), **p=0.01** | 1.7144 (-1.5892 to 6.9330), p=0.40 | **0.01** |
| **Mean clustering coefficient, estimated value (bootstrap-based 95% CI), p-value** | | | |
| Change from baseline to 1 year | -0.1316 (-0.2791 to -0.0107), **p=0.01** | 0.0162 (-0.0328 to 0.0926), p=0.70 | **0.03** |
| *2. Measures of nodal distance* | | | |
| **Mean shortest path, estimated value (bootstrap-based 95% CI), p-value** | | | |
| Change from baseline to 1 year | 0.7480 (0.3291 to 1.1559), **p=0.001** | -0.1682 (-0.3867 to -0.0180), **p=0.03** | **0.001** |
| **Global efficiency, estimated value (bootstrap-based 95% CI), p-value** | | | |
| Change from baseline to 1 year | -0.0911 (-0.2005 to -0.0049), **p=0.03** | 0.0226 (-0.0158 to 0.0850), p=0.40 | **0.02** |
| **Mean local efficiency, estimated value (bootstrap-based 95% CI), p-value** | | | |
| Change from baseline to 1 year | -0.1207 ( -0.2568 to -0.0102), **p=0.01** | 0.0177 (-0.0285 to 0.0893), p=0.60 | **0.03** |
| *3. Measures of network organisation* | | | |
| **Modularity coefficient (bootstrap-based 95% CI), p-value** | | | |
| Change from baseline to 1 year | 0.0340 (-0.0111 to 0.0784), p=0.20 | 0.0152 (-0.0126 to 0.0456), p=0.30 | 0.60 |

**Supplementary Table 5 (footnote).** Comparison between patients with and without McDonald MS conversion. See main text for full details on the methods used. Significant p-values are indicated with bold letters. *Abbreviations:* CI: confidence interval; CIS: clinically isolated syndrome; FU: follow-up; HCs: healthy controls.

## Supplementary Table 6. Estimated baseline and follow-up values of network parameters in patients with clinical MS conversion vs. HCs

| | Patients with clinical MS conversion | HCs | Patients with MS vs. HCs, estimated p-value |
|---|---|---|---|
| *1. Measures of nodal connectivity* | | | |
| **Mean nodal strength, estimated value (bootstrap-based 95% CI)** | | | |
| Baseline | 30.96 (21.13 to 42.96) | 24.08 (18.69 to 37.13) | 0.40 |
| Follow-up | 20.78 (16.41 to 30.08) | 23.50 (18.17 to 37.00) | 0.60 |
| **Mean clustering coefficient, estimated value (bootstrap-based 95% CI)** | | | |
| Baseline | 0.42 (0.28 to 0.62) | 0.31 (0.23 to 0.50) | 0.40 |
| Follow-up | 0.26 (0.20 to 0.40) | 0.30 (0.22 to 0.50) | 0.60 |
| *2. Measures of nodal distance* | | | |
| **Mean shortest path, estimated value (bootstrap-based 95% CI)** | | | |
| Baseline | 2.20 (1.76 to 2.66) | 2.46 (2.00 to 2.73) | 0.40 |
| Follow-up | 2.76 (2.34 to 3.07) | 2.49 (2.02 to 2.76) | 0.30 |
| **Global efficiency, estimated value (bootstrap-based 95% CI)** | | | |
| Baseline | 0.53 (0.42 to 0.68) | 0.45 (0.39 to 0.62) | 0.40 |
| Follow-up | 0.40 (0.35 to 0.52) | 0.45 (0.39 to 0.62) | 0.50 |
| **Mean local efficiency, estimated value (bootstrap-based 95% CI)** | | | |
| Baseline | 0.45 (0.31 to 0.63) | 0.34 (0.27 to 0.53) | 0.40 |
| Follow-up | 0.30 (0.24 to 0.43) | 0.34 (0.27 to 0.53) | 0.60 |
| *3. Measures of network organisation* | | | |
| **Modularity coefficient (bootstrap-based 95% CI)** | | | |
| Baseline | 0.06 (0.02 to 0.12) | 0.10 (0.08 to 0.13) | 0.30 |
| Follow-up | 0.09 (0.07 to 0.12) | 0.11 (0.09 to 0.14) | 0.40 |

**Supplementary Table 6 (footnote).** Comparison between patients with clinical MS conversion and HCs. See main text for full details on the methods used. Significant p-values are indicated with bold letters. *Abbreviations:* CI: confidence interval; CIS: clinically isolated syndrome; FU: follow-up; HCs: healthy controls.

## Supplementary Table 7. One-year changes in SCN parameters, in CIS patients with clinical MS conversion vs. HCs

| | Patients with clinical MS conversion | HCs | Patients with MS conversion vs. HCs, estimated p-value |
|---|---|---|---|
| ***1. Measures of nodal connectivity*** | | | |
| **Mean nodal strength, estimated value (bootstrap-based 95% CI), p-value** | | | |
| Change from baseline to 1 year | -10.18 (-16.22 to -0.87), **p=0.02** | -0.58 (-3.37 to 2.03), p=0.7 | 0.08 |
| **Mean clustering coefficient, estimated value (bootstrap-based 95% CI), p-value** | | | |
| Change from baseline to 1 year | -0.16 (-0.27 to -0.01), **p=0.02** | -0.01 (-0.05 to 0.04), p=0.8 | 0.08 |
| ***2. Measures of nodal distance*** | | | |
| **Mean shortest path, estimated value (bootstrap-based 95% CI), p-value** | | | |
| Change from baseline to 1 year | 0.56 (0.19 to 0.80), **p=0.001** | 0.03 (-0.06 to 0.13), p=0.6 | **0.001** |
| **Global efficiency, estimated value (bootstrap-based 95% CI), p-value** | | | |
| Change from baseline to 1 year | -0.12 (-0.20 to -0.02), **p=0.01** | $-1.46 * 10^{-4}$ (-0.03 to 0.03), p=0.99 | **0.02** |
| **Mean local efficiency, estimated value (bootstrap-based 95% CI), p-value** | | | |
| Change from baseline to 1 year | -0.15 (-0.25 to -0.01), **p=0.02** | -0.005 (-0.05 to 0.04), p=0.9 | 0.06 |
| ***3. Measures of network organisation*** | | | |
| **Modularity coefficient (bootstrap-based 95% CI), p-value** | | | |
| Change from baseline to 1 year | 0.03 (-0.02 to 0.07), p=0.5 | 0.0054 (-0.03 to 0.04), p=0.7 | 0.50 |

**Supplementary Table 7 (footnote).** Comparison between patients with clinical MS conversion and HCs. See main text for full details on the methods used. Significant p-values are indicated with bold letters. *Abbreviations:* CI: confidence interval; CIS: clinically isolated syndrome; FU: follow-up; HCs: healthy controls.

**Supplementary Table 8. Estimated baseline and follow-up values of network parameters in patients without clinical MS conversion vs. HCs**

| | Patients without clinical MS conversion | HCs | Patients without MS vs. HCs, estimated p-value |
|---|---|---|---|
| *1. Measures of nodal connectivity* | | | |
| **Mean nodal strength, estimated value (bootstrap-based 95% CI)** | | | |
| Baseline | 17.23 (13.98 to 23.02) | 24.08 (18.69 to 37.13) | 0.20 |
| Follow-up | 19.62 (15.22 to 26.99) | 23.50 (18.17 to 37.00) | 0.50 |
| **Mean clustering coefficient, estimated value (bootstrap-based 95% CI)** | | | |
| Baseline | 0.23 (0.19 to 0.30) | 0.31 (0.23 to 0.50) | 0.20 |
| Follow-up | 0.26 (0.21 to 0.36) | 0.30 (0.22 to 0.50) | 0.60 |
| *2. Measures of nodal distance* | | | |
| **Mean shortest path, estimated value (bootstrap-based 95% CI)** | | | |
| Baseline | 3.19 (2.82 to 3.50) | 2.46 (2.00 to 2.73) | **0.01** |
| Follow-up | 2.91 (2.51 to 3.26) | 2.49 (2.02 to 2.76) | 0.20 |
| **Global efficiency, estimated value (bootstrap-based 95% CI)** | | | |
| Baseline | 0.38 (0.34 to 0.45) | 0.45 (0.39 to 0.62) | 0.20 |
| Follow-up | 0.41 (0.36 to 0.50) | 0.45 (0.39 to 0.62) | 0.50 |
| **Mean local efficiency, estimated value (bootstrap-based 95% CI)** | | | |
| Baseline | 0.27 (0.23 to 0.34) | 0.34 (0.27 to 0.53) | 0.20 |
| Follow-up | 0.30 (0.25 to 0.40) | 0.34 (0.27 to 0.53) | 0.60 |
| *3. Measures of network organisation* | | | |
| **Modularity coefficient (bootstrap-based 95% CI)** | | | |
| Baseline | 0.10 (0.08 to 0.12) | 0.10 (0.08 to 0.13) | 0.70 |
| Follow-up | 0.10 (0.08 to 0.12) | 0.11 (0.09 to 0.14) | 0.80 |

**Supplementary Table 8 (footnote).** Comparison between patients without clinical MS conversion and HCs. See main text for full details on the methods used. Significant p-values are indicated with bold letters. *Abbreviations:* CI: confidence interval; CIS: clinically isolated syndrome; FU: follow-up; HCs: healthy controls;

**Supplementary Table 9. One-year changes in SCN parameters, in CIS patients without clinical MS conversion vs. HCs**

| | Patients without MS conversion | HCs | Patients with MS conversion vs. HCs, estimated p-value |
|---|---|---|---|
| ***1. Measures of nodal connectivity*** | | | |
| **Mean nodal strength, estimated value (bootstrap-based 95% CI), p-value** | | | |
| Change from baseline to 1 year | 2.39 (-1.03 to 7.52), p=0.3 | -0.58 (-3.37 to 2.03), p=0.7 | 0.25 |
| **Mean clustering coefficient, estimated value (bootstrap-based 95% CI), p-value** | | | |
| Change from baseline to 1 year | 0.03 (-0.02 to 0.11), p=0.4 | -0.01 (-0.05 to 0.04), p=0.8 | 0.40 |
| ***2. Measures of nodal distance*** | | | |
| **Mean shortest path, estimated value (bootstrap-based 95% CI), p-value** | | | |
| Change from baseline to 1 year | -0.28 (-0.58 to -0.07), **p=0.01** | 0.03 (-0.06 to 0.13), p=0.6 | **0.01** |
| **Global efficiency, estimated value (bootstrap-based 95% CI), p-value** | | | |
| Change from baseline to 1 year | 0.03 (-0.01 to 0.09), p=0.2 | $-1.46 * 10^{-4}$ (-0.03 to 0.03), p=0.99 | 0.30 |
| **Mean local efficiency, estimated value (bootstrap-based 95% CI), p-value** | | | |
| Change from baseline to 1 year | 0.03 (-0.02 to 0.10), p=0.3 | -0.005 (-0.05 to 0.04), p=0.9 | 0.40 |
| ***3. Measures of network organisation*** | | | |
| **Modularity coefficient (bootstrap-based 95% CI), p-value** | | | |
| Change from baseline to 1 year | 0.01 (-0.02 to 0.03), p=0.8 | 0.0054 (-0.03 to 0.04), p=0.7 | 0.99 |

**Supplementary Table 9 (footnote).** Comparison between patients without clinical MS conversion and HCs. See main text for full details on the methods used. Significant p-values are indicated with bold letters. *Abbreviations:* CI: confidence interval; CIS: clinically isolated syndrome; FU: follow-up; HCs: healthy controls.

## Supplementary Table 10. Estimated baseline and follow-up values of network parameters using cortical thickness data not adjusted for lesion load, in all CIS patients and controls

| | CIS patients (all) | HCs | Patients vs. HCs, estimated p-value |
|---|---|---|---|
| *1. Measures of nodal connectivity* | | | |
| **Mean nodal strength, estimated value (bootstrap-based 95% CI)** | | | |
| Baseline | 20.0565 (14.1787 to 30.6859) | 24.1688 (18.9864 to 37.8468) | 0.50 |
| Follow-up | 14.5305 (12.1800 to 18.0834) | 23.6680 (18.7561 to 37.0835) | **0.01** |
| **Mean clustering coefficient, estimated value (bootstrap-based 95% CI)** | | | |
| Baseline | 0.2965 (0.2199 to 0.4469) | 0.3043 (0.2325 to 0.5111) | 0.99 |
| Follow-up | 0.2082 (0.1801 to 0.2482) | 0.2988 (0.2277 to 0.4972) | **0.04** |
| *2. Measures of nodal distance* | | | |
| **Mean shortest path, estimated value (bootstrap-based 95% CI)** | | | |
| Baseline | 3.1146 (2.4741 to 3.7114) | 2.4546 (1.9757 to 2.7074) | 0.10 |
| Follow-up | 3.7447 (3.3611 to 4.0891) | 2.4765 (2.0007 to 2.7208) | **<0.001** |
| **Global efficiency, estimated value (bootstrap-based 95% CI)** | | | |
| Baseline | 0.4125 (0.3520 to 0.5301) | 0.4566 (0.3974 to 0.6280) | 0.50 |
| Follow-up | 0.3507 (0.3183 to 0.3898) | 0.4534 (0.3923 to 0.6209) | **0.01** |
| **Mean local efficiency, estimated value (bootstrap-based 95% CI)** | | | |
| Baseline | 0.3264 (0.2540 to 0.4662) | 0.3438 (0.2758 to 0.5395) | 0.90 |
| Follow-up | 0.2445 (0.2154 to 0.2846) | 0.3390 (0.2715 to 0.5269) | **0.03** |
| *3. Measures of network organisation* | | | |
| **Modularity coefficient (bootstrap-based 95% CI)** | | | |
| Baseline | 0.0722 (0.0284 to 0.0983) | 0.1072 (0.0829 to 0.1367) | 0.08 |
| Follow-up | 0.1052 (0.0906 to 0.1242) | 0.1050 (0.0840 to 0.1335) | 0.99 |

**Supplementary Table 10 (footnote).** Comparison between CIS patients and HCs using cortical thickness data not adjusted for lesion load but adjusted for age, gender and mean cortical thickness. See main text for full details on the methods used. Significant p-values are indicated with bold letters. *Abbreviations:* CI: confidence interval; CIS: clinically isolated syndrome; FU: follow-up; HCs: healthy controls;

**Supplementary Table 11. One-year changes in SCN parameters using cortical thickness data non-adjusted for lesion load, in all CIS patients and controls**

| | CIS patients (all) | HCs | Patients vs. HCs, estimated p-value |
|---|---|---|---|
| *1. Measures of nodal connectivity* | | | |
| **Mean nodal strength, estimated value (bootstrap-based 95% CI), p-value** | | | |
| Change from baseline to 1 year | -5.5260 (-16.2191 to 1.4599), p=0.25 | -0.5007 (-3.3987 to 1.6976), p=0.70 | 0.30 |
| **Mean clustering coefficient, estimated value (bootstrap-based 95% CI), p-value** | | | |
| Change from baseline to 1 year | -0.0883 (-0.2403 to 0.0046), p=0.08 | -0.0055 (-0.0538 to 0.0320), p=0.80 | 0.20 |
| *2. Measures of nodal distance* | | | |
| **Mean shortest path, estimated value (bootstrap-based 95% CI), p-value** | | | |
| Change from baseline to 1 year | 0.6301 (-0.0234 to 1.3032), p=0.08 | 0.0219 (-0.0565 to 0.1155), p=0.60 | 0.10 |
| **Global efficiency, estimated value (bootstrap-based 95% CI), p-value** | | | |
| Change from baseline to 1 year | -0.0618 (-0.1777 to 0.0168), p=0.15 | -0.0032 (-0.0353 to 0.0230), p=0.90 | 0.25 |
| **Mean local efficiency, estimated value (bootstrap-based 95% CI), p-value** | | | |
| Change from baseline to 1 year | -0.0819 (-0.2228 to 0.0069), p=0.09 | -0.0048 (-0.0488 to 0.0285), p=0.80 | 0.20 |
| *3. Measures of network organisation* | | | |
| **Modularity coefficient (bootstrap-based 95% CI), p-value** | | | |
| Change from baseline to 1 year | 0.0330 (0.0019 to 0.0763), p=0.05 | -0.0023 (-0.0279 to 0.0256), p=0.90 | 0.20 |

**Supplementary Table 11 (footnote).** Comparison between all CIS patients and HCs using cortical thickness data not adjusted for lesion load but adjusted for age, gender and mean cortical thickness. See main text for full details on the methods used. Significant p-values are indicated with bold letters. *Abbreviations:* CI: confidence interval; CIS: clinically isolated syndrome; FU: follow-up; HCs: healthy controls;

**Supplementary Table 12. Estimated baseline and follow-up values of network parameters using cortical thickness data non-adjusted for lesion load, in patients with and without clinical MS conversion**

| | Patients with clinical MS conversion | Patients without clinical MS conversion | Patients with vs. without MS conversion, estimated p-value |
|---|---|---|---|
| *1. Measures of nodal connectivity* | | | |
| **Mean nodal strength, estimated value (bootstrap-based 95% CI)** | | | |
| Baseline | 30.8093 (21.3159 to 42.3899) | 17.3398 (13.9670 to 23.2754) | **0.02** |
| Follow-up | 19.8222 (15.1510 to 28.5493) | 18.6040 (15.0450 to 25.2021) | 0.80 |
| **Mean clustering coefficient, estimated value (bootstrap-based 95% CI)** | | | |
| Baseline | 0.4203 (0.2797 to 0.6070) | 0.2301 (0.1924 to 0.3019) | **0.02** |
| Follow-up | 0.2550 (0.2013 to 0.3738) | 0.2486 (0.2082 to 0.3305) | 0.90 |
| *2. Measures of nodal distance* | | | |
| **Mean shortest path, estimated value (bootstrap-based 95% CI)** | | | |
| Baseline | 2.2001 (1.7755 to 2.6394) | 3.1743 (2.7928 to 3.5029) | **<0.001** |
| Follow-up | 2.8508 (2.4404 to 3.2099) | 3.0401 (2.6569 to 3.3745) | 0.50 |
| **Global efficiency, estimated value (bootstrap-based 95% CI)** | | | |
| Baseline | 0.5250 (0.4224 to 0.6674) | 0.3762 (0.3379 to 0.4408) | **0.02** |
| Follow-up | 0.4036 (0.3486 to 0.5107) | 0.3930 (0.3505 to 0.4717) | 0.90 |
| **Mean local efficiency, estimated value (bootstrap-based 95% CI)** | | | |
| Baseline | 0.4465 (0.3164 to 0.6202) | 0.2677 (0.2300 to 0.3372) | **0.02** |
| Follow-up | 0.2935 (0.2395 to 0.4085) | 0.2860 (0.2452 to 0.3664) | 0.90 |
| *3. Measures of network organisation* | | | |
| **Modularity coefficient (bootstrap-based 95% CI)** | | | |
| Baseline | 0.0610 (0.0203 to 0.1186) | 0.0901 (0.0750 to 0.1111) | 0.60 |
| Follow-up | 0.0976 (0.0784 to 0.1195) | 0.0983 (0.0831 to 0.1196) | 0.99 |

**Supplementary Table 12 (footnote).** Comparison between patients with and without clinical MS conversion using cortical thickness data not adjusted for lesion load but adjusted for age, gender and mean cortical thickness. See main text for full details on the methods used. Significant p-values are indicated with bold letters. *Abbreviations:* CI: confidence interval; CIS: clinically isolated syndrome; FU: follow-up; HCs: healthy controls.

## Supplementary Table 13. One-year changes in SCN parameters using cortical thickness data non-adjusted for lesion load, in CIS patients with and without clinical MS conversion

| | Patients with clinical MS conversion | Patients without clinical MS conversion | Patients with vs. without MS conversion, estimated p-value |
|---|---|---|---|
| ***1. Measures of nodal connectivity*** | | | |
| **Mean nodal strength, estimated value (bootstrap-based 95% CI), p-value** | | | |
| Change from baseline to 1 year | -10.9871 (-19.5218 to -1.9508), **p=0.005** | 1.2642 (-1.2912 to 4.7118), p=0.40 | **0.005** |
| **Mean clustering coefficient, estimated value (bootstrap-based 95% CI), p-value** | | | |
| Change from baseline to 1 year | -0.1653 (-0.3062 to -0.0221), **p=0.01** | 0.0185 (-0.0201 to 0.0658), p=0.40 | **0.01** |
| ***2. Measures of nodal distance*** | | | |
| **Mean shortest path, estimated value (bootstrap-based 95% CI), p-value** | | | |
| Change from baseline to 1 year | 0.6506 (0.2800 to 0.9909), **p<0.001** | -0.1342 (-0.3324 to 0.0345), p=0.15 | **<0.001** |
| **Global efficiency, estimated value (bootstrap-based 95% CI), p-value** | | | |
| Change from baseline to 1 year | -0.1214 (-0.2257 to -0.0211), **p=0.005** | 0.0169 (-0.0165 to 0.0599), p=0.40 | **0.005** |
| **Mean local efficiency, estimated value (bootstrap-based 95% CI), p-value** | | | |
| Change from baseline to 1 year | -0.1530 (-0.2836 to -0.0221), **p=0.01** | 0.0183 (-0.0187 to 0.0637), p=0.40 | **0.01** |
| ***3. Measures of network organisation*** | | | |
| **Modularity coefficient (bootstrap-based 95% CI), p-value** | | | |
| Change from baseline to 1 year | 0.0366 (-0.0177 to 0.0844), p=0.30 | 0.0082 (-0.0155 to 0.0317), p=0.50 | 0.50 |

**Supplementary Table 13 (footnote).** Comparison between patients with and without clinical MS conversion using cortical thickness data not adjusted for lesion load but adjusted for age, gender and mean cortical thickness. See main text for full details on the methods used. Significant p-values are indicated with bold letters. *Abbreviations:* CI: confidence interval; CIS: clinically isolated syndrome; FU: follow-up; HCs: healthy controls.

**Supplementary Table 14. Estimated baseline and follow-up values of network parameters in patients with supratentorial lesions only and supra- and infratentorial lesions**

| | Patients with infra- and supratentorial lesions | Patients with supratentorial lesions only | Patients with supratentorial lesions only vs. infra- and supratentorial lesions, estimated p-value |
|---|---|---|---|
| *1. Measures of nodal connectivity* | | | |
| **Mean nodal strength, estimated value (bootstrap-based 95% CI)** | | | |
| Baseline | 31.7903 (22.2722 to 43.3564) | 20.4792 (16.1678 to 29.2595) | 0.08 |
| Follow-up | 21.3210 ( 15.4187 to 29.6407) | 20.8007 (16.5693 to 29.0357) | 0.99 |
| **Mean clustering coefficient, estimated value (bootstrap-based 95% CI)** | | | |
| Baseline | 0.4278 ( 0.2831 to 0.6141) | 0.2630 (0.2144 to 0.3836) | 0.10 |
| Follow-up | 0.2736 ( 0.2035 to 0.401) | 0.2723 ( 0.3849 to 0.2231) | 0..99 |
| *2. Measures of nodal distance* | | | |
| **Mean shortest path, estimated value (bootstrap-based 95% CI)** | | | |
| Baseline | 2.1400 ( 1.7628 to 2.5576) | 2.7737 (2.3710 to 3.0871) | **0.03** |
| Follow-up | 2.6601 ( 2.3428 to 3.0293) | 2.7648 (2.3808 to 3.0814) | 0.80 |
| **Global efficiency, estimated value (bootstrap-based 95% CI)** | | | |
| Baseline | 0.5360 ( 0.4242 to 0.6807) | 0.4094 (0.3603 to 0.5160) | 0.10 |
| Follow-up | 0.4296 ( 0.3603 to 0.5280) | 0.4190 (0.3713 to 0.5227) | 0.90 |
| **Mean local efficiency, estimated value (bootstrap-based 95% CI)** | | | |
| Baseline | 0.4549 ( 0.3196 to 0.6298) | 0.3012 (0.2520 to 0.4170) | 0.10 |
| Follow-up | 0.3140 ( 0.2448 to 0.4334) | 0.3105 (0.2622 to 0.4202) | 0.99 |
| *3. Measures of network organisation* | | | |
| **Modularity coefficient (bootstrap-based 95% CI)** | | | |
| Baseline | 0.0613 ( 0.0188 to 0.1192) | 0.1000 (0.0822 to 0.1240) | 0.40 |
| Follow-up | 0.1117 ( 0.0923 to 0.1398) | 0.1017 (0.0844 to 0.1244) | 0.60 |

**Supplementary Table 14 (footnote).** Comparison between patients with supratentorial lesions only and supra- and infratentorial lesions. See main text for full details on the methods used. Significant p-values are indicated with bold letters. *Abbreviations:* CI: confidence interval; CIS: clinically isolated syndrome; FU: follow-up; HCs: healthy controls;

## Supplementary Table 15. One-year changes in SCN parameters, in CIS patients with supratentorial lesions only and supra- and infratentorial lesions

| | Patients with infra- and supratentorial lesions | Patients with supratentorial lesions only | Patients with supratentorial lesions only vs. infra- and supratentorial lesions, estimated p-value |
|---|---|---|---|
| ***1. Measures of nodal connectivity*** | | | |
| **Mean nodal strength, estimated value (bootstrap-based 95% CI), p-value** | | | |
| Change from baseline to 1 year | -10.4693 (-19.0404 to -1.6575), **p=0.01** | 0.3214 (-1.5832 to 2.5635), p=0.80 | **0.01** |
| **Mean clustering coefficient, estimated value (bootstrap-based 95% CI), p-value** | | | |
| Change from baseline to 1 year | -0.1542 (-0.2957 to -0.0122), **p=0.02** | 0.0094 (-0.0246 to 0.0453), p=0.60 | **0.02** |
| ***2. Measures of nodal distance*** | | | |
| **Mean shortest path, estimated value (bootstrap-based 95% CI), p-value** | | | |
| Change from baseline to 1 year | 0.5201 (0.1892 to 0.8413), **p=0.001** | -0.0089 (-0.0974 to 0.0735), p=0.90 | **0.001** |
| **Global efficiency, estimated value (bootstrap-based 95% CI), p-value** | | | |
| Change from baseline to 1 year | -0.1064 (-0.2108 to -0.0047), **p=0.02** | 0.0096 (-0.0146 to 0.0352), p=0.50 | **0.02** |
| **Mean local efficiency, estimated value (bootstrap-based 95% CI), p-value** | | | |
| Change from baseline to 1 year | -0.1409 (-0.2711 to -0.0113), **p=0.02** | 0.0094 (-0.0215 to 0.0423), p=0.60 | **0.02** |
| ***3. Measures of network organisation*** | | | |
| **Modularity coefficient (bootstrap-based 95% CI), p-value** | | | |
| Change from baseline to 1 year | 0.0505 ($-1.2236*10^{-5}$ to 0.0950), p=0.055 | 0.0017 (-0.0246 to 0.0266), p=0.90 | 0.20 |

**Supplementary Table 15 (footnote).** Comparison between patients with supratentorial lesions only and supra- and infratentorial lesions. See main text for full details on the methods used. Significant p-values are indicated with bold letters. *Abbreviations:* CI: confidence interval; CIS: clinically isolated syndrome; FU: follow-up; HCs: healthy controls.

# SUPPLEMENTARY FIGURES AND FIGURE LEGENDS

## Supplementary figure 1. Network parameters at baseline in all CIS patients and HCs

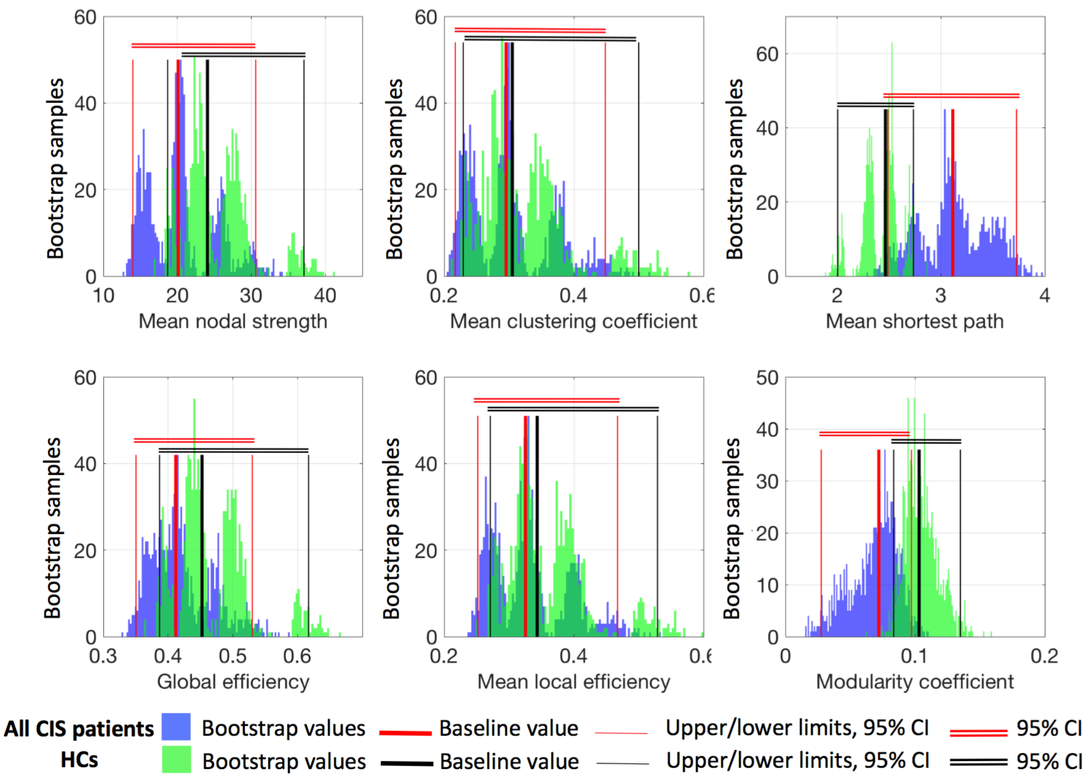


**Supplementary figure 1.** (Figure legend) This figure shows the baseline values of all network parameters for all CIS patients (red) and HCs. None of the parameters was significantly different between all CIS and HCs' networks. See **Supplementary Table 2** for more details. *Abbreviations:* CIS: clinically isolated syndrome; HC: healthy control; 95% CI: 95% Confidence Interval.

**Supplementary figure 2. Changes in network parameters over 1 year for all CIS patients and HCs**

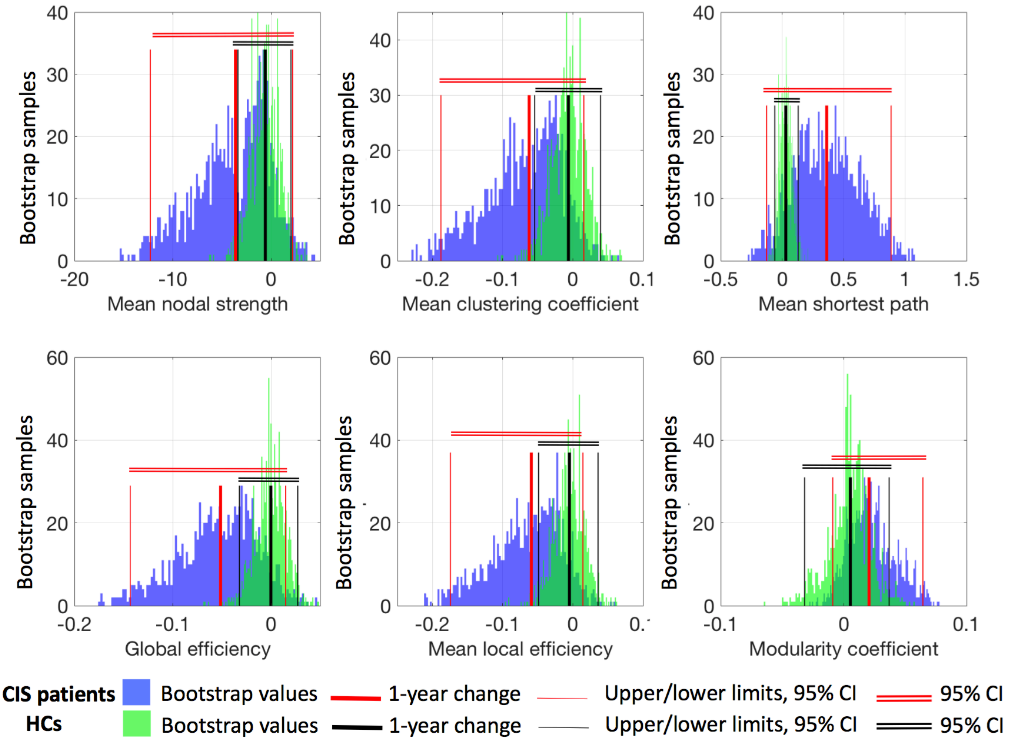


**Supplementary figure 2.** (Figure legend) This figure shows the point estimates for the changes in network parameters and the bootstrap-based 95% CIs for those changes, for the network of all CIS patients (red) and the HC network (black). The 95% CI were estimated from the bootstrap distributions, for each group and each parameter. As can be seen, the HC network only shows minimal changes over time, whereas the changes in the CIS network often depart from the null value (i.e. from zero). However, neither the CIS nor the HC networks show statistically significant changes over 1 year, since bootstrap-based 95% CIs always include the null value. See **Table 2** for more details. *Abbreviations:* CIS: clinically isolated syndrome; CI: Confidence Interval; HCs: healthy controls.

**Supplementary figure 3. Network parameters at baseline in CIS patients with and without conversion to MS**

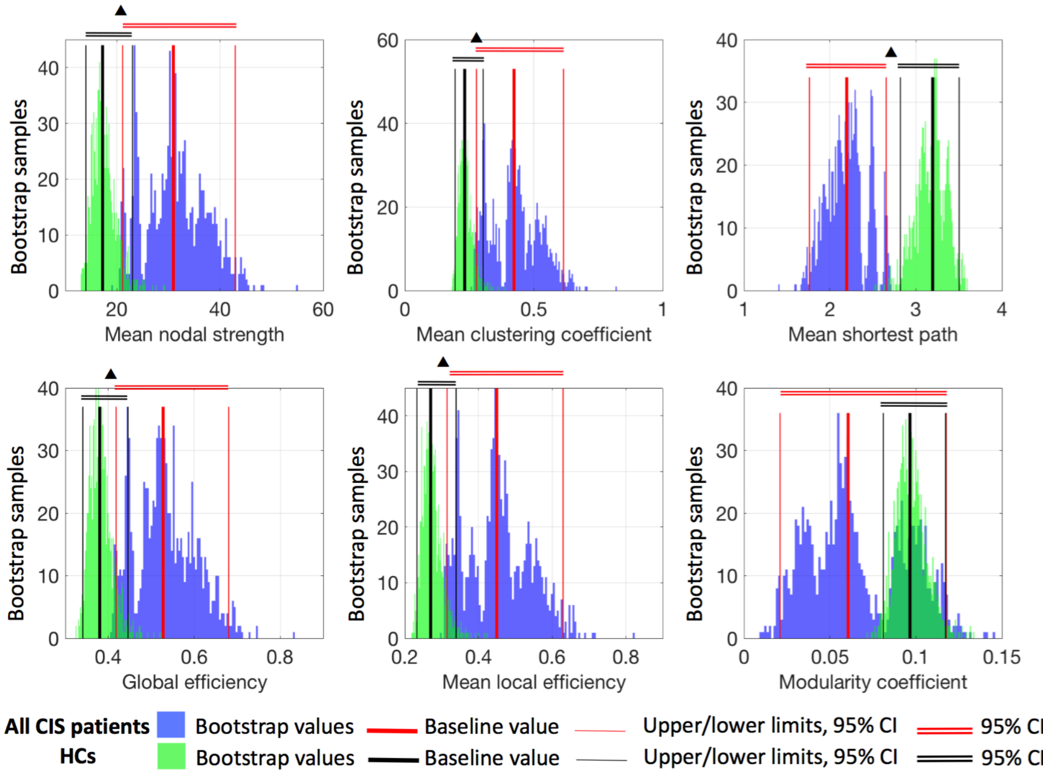


**Supplementary figure 3.** (Figure legend) ▲: significant difference between patients and controls. This figure shows the baseline values of all network parameters for all CIS patients who convert to MS (red) and those who do not convert (black). As can be seen, 95% CIs overlap for most parameters, except for mean shortest path, which is *significantly* smaller in converters than in non-converters. See **Supplementary Table 3** for more details. *Abbreviations:* CIS: clinically isolated syndrome; HC: healthy control; 95% CI: 95% Confidence Interval.

**Supplementary figure 4. Changes in network parameters over 1 year for CIS patients with and without conversion to MS**

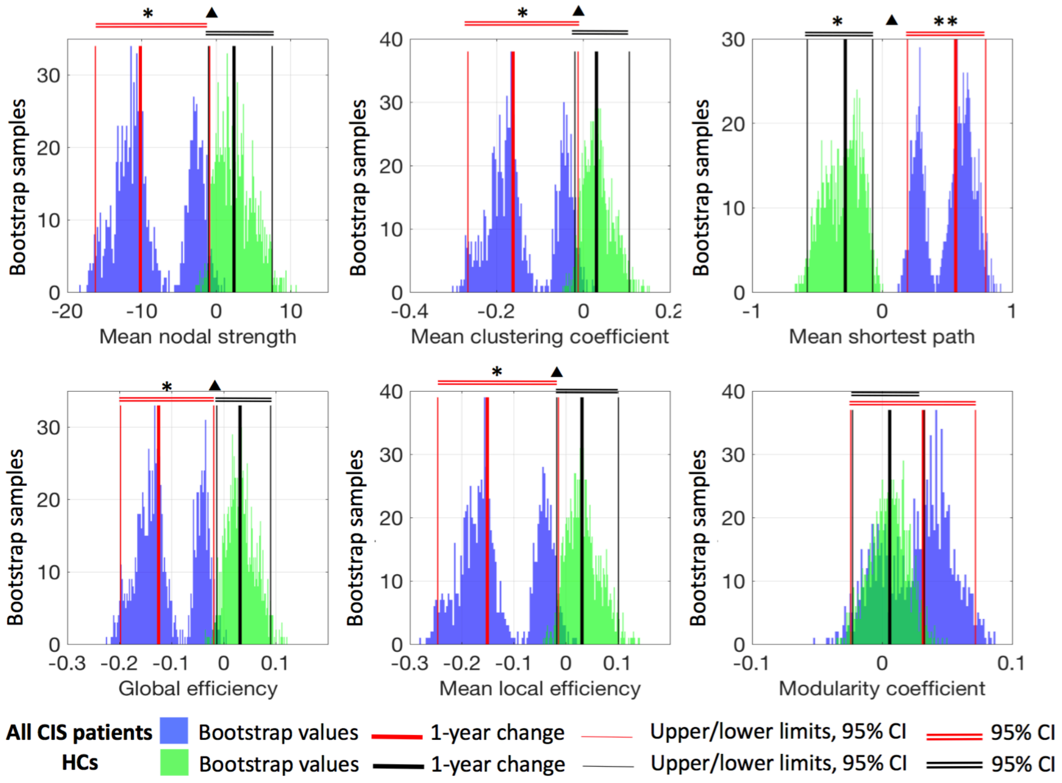


**Supplementary figure 4.** (Figure legend) ▲: significant difference between patients and controls; *: significant change over time ($p<0.05$); **: significant change over time ($p<0.01$). This figure shows the point estimates for the changes in network parameters and the bootstrap-based 95% CIs for those changes, for the network of CIS patients who convert to MS (red) and the network of those CIS patients who do not convert (black). The 95% CI were estimated from the bootstrap distributions, for each group and each parameter. As can be seen, the non-converter network only shows minimal changes over time, mirroring HC network changes, whereas the changes in the *converter network* often depart from the null value (i.e. from zero). Importantly, changes in the converter network are significant for most of the parameters, i.e. mean nodal strength, clustering coefficient, mean shortest path, global and local efficiency, where the bootstrap-based 95% CIs do not include the null value. See **Table 3** for more details. *Abbreviations:* CIS: clinically isolated syndrome; CI: Confidence Interval.
